# Surface-emitting ring quantum cascade lasers

**DOI:** 10.1515/nanoph-2025-0248

**Published:** 2025-08-28

**Authors:** Rolf Szedlak, Benedikt Schwarz, Gottfried Strasser

**Affiliations:** 27259 Institute of Solid State Electronics, TU Wien, Vienna, Austria

**Keywords:** quantum cascade laser, ring cavity, surface emission, distributed feedback grating, emission beam, sensing

## Abstract

This review highlights the development and diverse applications of surface-emitting ring quantum cascade lasers. These lasers, featuring a ring-shaped cavity and a second-order distributed feedback grating, enable controlled surface emission, stable single-mode operation, and collimated, circularly symmetric beams. These advancements have expanded their use in scientific and industrial applications, with significant progress in extending their operation from the mid-infrared to the terahertz regime. Innovations such as microring designs, phase-shifted gratings and buried heterostructures have enhanced their scalability, beam quality, and suitability for continuous-wave operation at room temperature, unlocking opportunities in spectroscopy, imaging, and communication. On-chip optical solutions and integrated architectures combining emission and detection within a single chip further pave the way for compact and efficient systems for remote sensing and gas monitoring. Progress in miniaturization, thermal stability, and wavelength consistency strengthens the role of ring-based lasers in industrial, environmental, and medical diagnostics. These advancements, alongside scalable fabrication techniques and enhanced modulation schemes, position ring quantum cascade lasers as key enablers for the next generation of robust, energy-efficient sensors tailored to diverse scientific and industrial needs.

## 1 Introduction

The invention of the quantum cascade laser (QCL) by Faist et al. in 1994 marked a pivotal moment in the development of mid-infrared (MIR) and terahertz (THz) semiconductor lasers [1]. Unlike traditional interband lasers, which are limited by material bandgaps, QCLs leverage intersubband transitions within quantum wells [2], [3], enabling operation across a wide range of wavelengths by engineering the layer structure. This breakthrough opened up new possibilities for applications requiring precise MIR and THz emission, such as gas sensing [4], [5], [6], [7], environmental monitoring [8], [9], [10], and medical diagnostics [11], [12], [13], [14], [15], [16], which benefit from the unique ability of QCLs to target specific absorption lines of molecules. A conventional QCL typically employs a straight ridge-type cavity with two cleaved facets, a design that offers several advantages, including straightforward fabrication, high optical confinement, robustness, and high output power. However, this configuration also exhibits some limitations: the cleaved facets are defined only during the final stages of fabrication, preventing on-chip testing and device optimization before completion. Additionally, the small emitting area of the cleaved facet leads to a highly divergent and asymmetric emission pattern, which can complicate beam shaping and integration in optical systems. To address these limitations, alternative laser cavity geometries have been explored since the early development of QCL technology. Among these, a circular cavity design was first demonstrated in 1996 by Faist et al. in the form of a QCL disk laser [17], a geometry that has also been successfully implemented in other active laser materials [18], [19].

For QCLs in particular, the circular geometry introduces distinct advantages, such as a reduction of radiative losses through inherent transverse magnetic (TM) polarization, which suppresses vertical emission, thus enhancing optical confinement within the circular laser cavity. Furthermore, the unipolar nature of carrier transport in QCLs minimizes surface recombination losses. The use of longer emission wavelengths in QCLs also facilitates single-mode operation in microcavity lasers, a feature first demonstrated by Gmachl et al. in 1997, showcasing the potential of the circular geometry for achieving narrow linewidths and stable single-frequency output [20], [21]. These microdisk lasers achieve light output primarily via evanescent coupling in the horizontal plane, but the pedestal supporting the microdisk often induces additional light emission in non-horizontal directions. Such emission is not well-controlled, limiting the predictability and directionality of the output.

Achieving controllable surface emission is crucial for applications requiring small beam divergence and a symmetric beam profile, characteristics typically associated with vertical cavity surface-emitting lasers (VCSELs) [22], [23], [24], [25]. However, due to the intersubband selection rules in QCLs [26], which inherently favor TM polarization, the realization of VCSEL-like structures in QCLs is not feasible. Nevertheless, several strategies for generating surface emission in QCLs have been developed. For instance, photonic crystal (PC) QCLs demonstrated by Colombelli et al. in 2003 utilized periodic structures to provide feedback and diffract light vertically [27]. Similarly, second-order distributed feedback (DFB) gratings have been employed to achieve surface emission in linear QCLs, as demonstrated by Hofstetter et al. in 1999 on InP [28] and Schrenk et al. in 2000 on GaAs [29]. However, integrating DFB gratings with cleaved facets often leads to challenges in controlling the phase relation between grating and facets, which not only influences but can even degrade performance [30]. This issue can be addressed by using etched facets [31], [32] or adopting a geometry that eliminates the need for facets altogether, such as a ring cavity.

A combination of a ring cavity with a second-order DFB grating for controlled surface emission was first demonstrated by Mujagic et al. in 2008 [33]. This innovative approach paved the way for extensive research into ring QCLs, leading to significant improvements in device performance and expanding their range of applications. This review will explore the various developments, optimizations, and applications of ring QCLs, characterized by their distinctive ring-shaped cavity and second-order DFB grating for surface emission.

## 2 Surface emission

A fundamental element that enables surface emission in ring QCLs is the second-order DFB grating. It converts guided waveguide modes that typically propagate in-plane into radiation emitted perpendicular to the chip surface. This mode conversion is essential for achieving surface-emitting configurations that deliver circularly symmetric beams with tailored polarization properties. The core principle of a DFB grating is governed by the Bragg condition [34], which determines the specific wavelength for which the grating provides strong, wavelength-selective optical feedback through constructive interference. It reads

(1) $$\lambda_{B}=\frac{2\Lambda n_{\text{eff}}}{m_{\text{DFB}}}.$$

**Figure 1:**
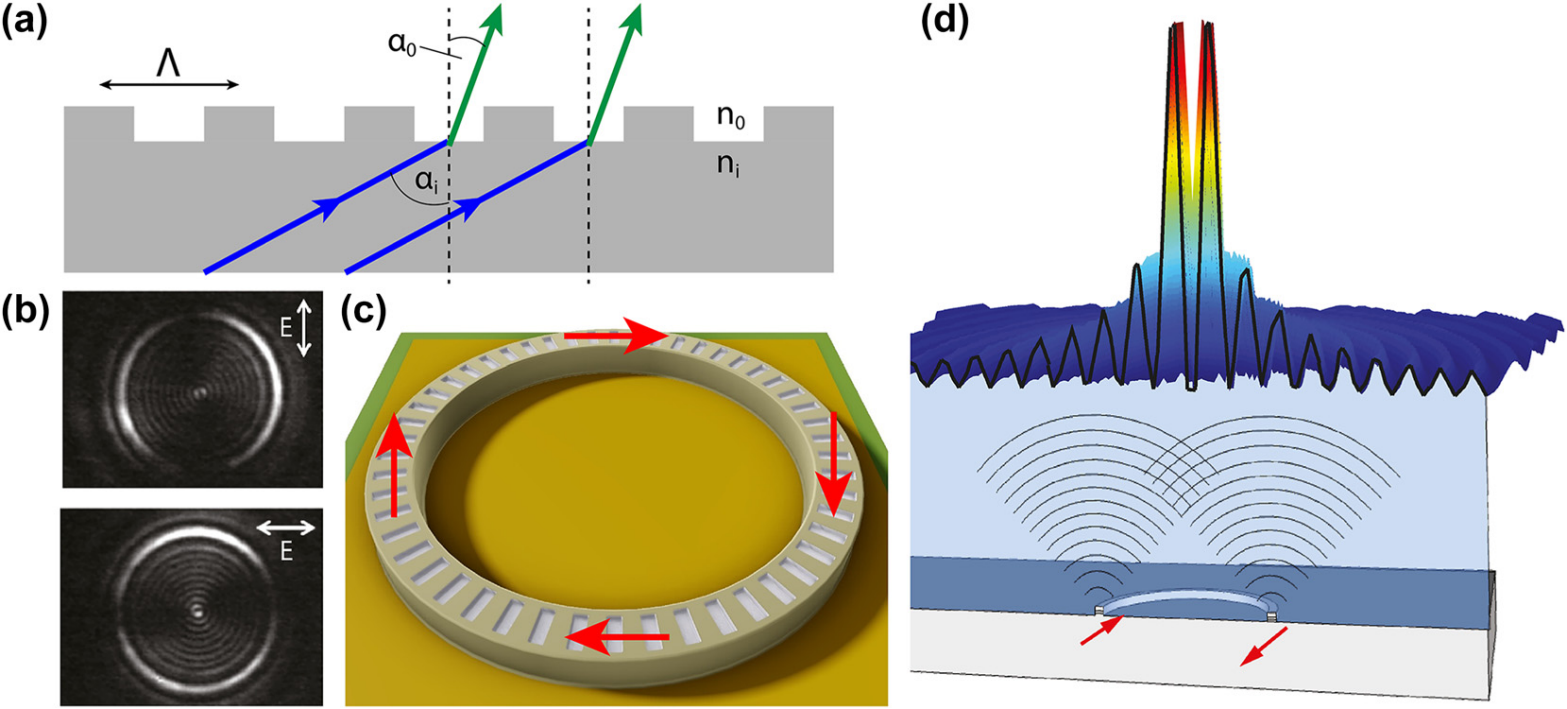
Surface emission and interference in ring QCLs. (a) Schematic illustration of the grating equation, showing diffraction for arbitrary angles of incidence and emission across two different media. (b) Measured nearfield polarization pattern of a ring QCL, confirming azimuthal polarization; the top panel shows vertical polarization, while the bottom panel shows horizontal polarization. (c) Schematic representation of the nearfield electric field vectors around the ring, showing anti-parallel orientation at opposing sides. (d) Analogy between a ring QCL and a two-dimensional double-slit setup, highlighting the phase relationship that leads to a central intensity minimum in the farfield. (b) Is reprinted from Ref. [37], with permission. ©2017 Rolf Szedlak, All Rights reserved; (d) is reprinted from Ref. [38], with permission. ©2014 Clemens Schwarzer, All Rights reserved.

Here, *λ*_*B*_ is the Bragg wavelength, Λ the grating period, *n*_eff_ the effective refractive index, and *m*_DFB_ the grating order. More generally, the grating equation relates incident and diffracted angles for media with refractive indices *n*_*i*_ and *n*_*o*_ [35], [36]:

(2) $$\Lambda \left[n_{i}\sin\left(\alpha_{i}\right)\pm n_{o}\sin\left(\alpha_{o}\right)\right]=m_{\text{diff}}\lambda_{B}$$

with *α*_*i*_ and *α*_*o*_ denoting the angles relative to the grating normal of the incoming and outgoing waves, respectively, and *m*_diff_ referring to the diffraction order. Figure 1(a) illustrates both the parameters involved and the working principle underlying the grating equation.

In typical DFB laser configurations, the guided mode propagates perpendicular to the grating normal (*α*_*i*_ = 90°), and *n*_*i*_ = *n*_eff_. Combining Eq. (1) and Eq. (2) yields an explicit expression for the emission angle of the outgoing wave:

(3) $$\alpha_{o}=\arcsin\left[\frac{n_{\text{eff}}}{n_{o}}\left(\frac{2m_{\text{diff}}}{m_{\text{DFB}}}\mp 1\right)\right].$$

For a first-order DFB grating (*m*_DFB_ = 1), Eq. (3) simplifies to *α*_*o*_ = ±90° for *m*_diff_ = 1, indicating in-plane propagation only. In contrast, a second-order DFB grating (*m*_DFB_ = 2) allows additional diffraction orders. Specifically, the first diffraction order yields *α*_*o*_ = 0° ± 180°, enabling vertical emission normal to the chip surface. This vertical emission is independent of *n*_*o*_, supporting both surface and substrate emission. Meanwhile, the second diffraction order at *α*_*o*_ = ±90° provides the necessary wavelength-selective optical feedback. Thus, the second-order DFB grating simultaneously enforces single-mode operation and enables out-of-plane emission.

Beyond wavelength selection and mode conversion, second-order gratings also shape the emitted beam’s polarization. Due to the intersubband selection rule [26], the optical transitions in QCLs involve electric fields oriented along the growth direction. When light is refracted by 90° at the grating, this electric field rotates correspondingly, producing an azimuthally polarized emission beam. The polarization-resolved nearfield measurements in Figure 1(b) confirm this behavior, showing the characteristic azimuthal pattern [39]. Figure 1(c) schematically illustrates the orientation of the electric field vectors around the ring, with anti-parallel directions on opposite sides, corresponding to a *π* phase shift.

This phase relationship is critical for understanding the ring’s farfield emission pattern. The ring QCL can be viewed as a two-dimensional analog of the classical double-slit experiment, as depicted in Figure 1(d). In the classical double-slit experiment, in-phase wavefronts from both slits interfere constructively at the center, resulting in a central intensity maximum. In contrast, the *π* phase shift between opposing sides of the ring leads to destructive interference at the center, yielding a characteristic central intensity minimum surrounded by concentric interference fringes in the farfield pattern.

These theoretical considerations explain how second-order DFB gratings achieve efficient mode conversion from guided in-plane modes to vertically emitted, azimuthally polarized beams, enabling the unique surface-emitting properties of ring QCLs.

## 3 Operational performance

**Figure 2:**
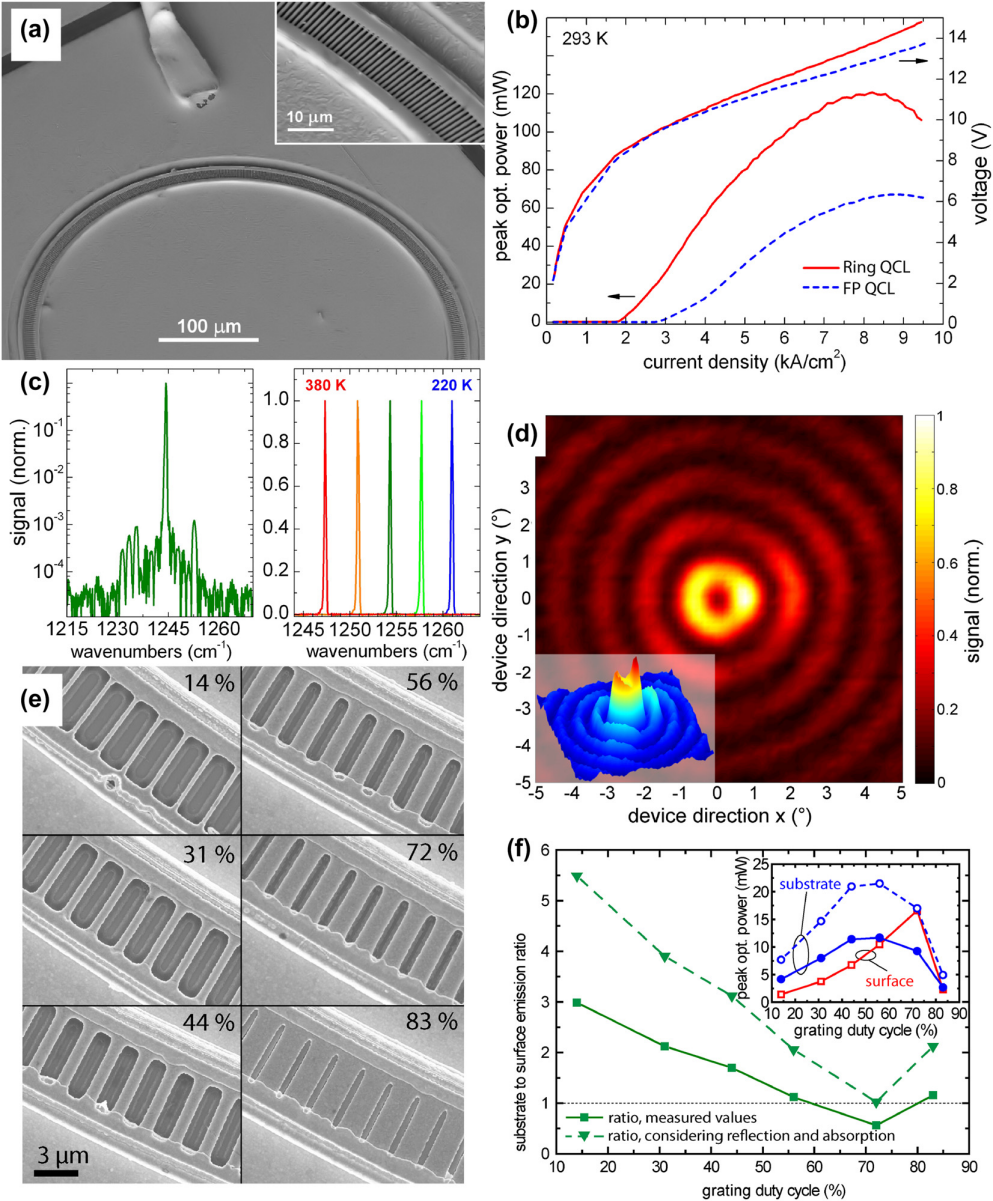
Performance and emission characteristics of ring QCLs. (a) SEM image of the first ring QCL featuring a circular laser cavity with an integrated second-order DFB grating. The inset provides a magnified view of the grating structure. (b) Comparison of the light-current-voltage (LIV) characteristics of a ring QCL and a straight FP QCL. The ring QCL exhibits a lower threshold current density and a higher peak optical power. (c) Single-mode spectrum of a ring QCL with an SMSR of 30 dB (left) and temperature-tuning characteristics indicating a tuning coefficient of −0.085 cm^−1^/K (right). (d) Farfield pattern of a ring QCL with concentric interference rings and a central intensity minimum. The inset depicts a 3D representation of this farfield. (e) SEM images of ring QCLs with different grating duty cycles (GDCs). (f) Substrate-to-surface emission ratio as a function of the GDC. The inset shows the peak optical power of surface and substrate emission as a function of the GDC. Considering reflections and absorption, the substrate emission shows stronger emission for all GDCs. (a) Is reprinted from Ref. [33], with permission from AIP Publishing; (b–c) are reprinted from Ref. [40], with permission from AIP Publishing; (d) is adapted from Ref. [40], with permission from AIP Publishing; (e–f) are reprinted from Ref. [41], with permission from AIP Publishing.

The performance of ring QCLs has evolved significantly since their initial development. The first ring QCLs, reported by Mujagic et al. in 2008, operated at cryogenic temperatures with an emission wavelength around 4 µm. A scanning electron microscopy (SEM) image of the first ring QCL is provided in Figure 2(a).

These devices operated in pulsed mode, delivering a peak optical output power of approximately 70 mW and a threshold current density of 4.0 kA/cm^2^ [33]. Despite their successful demonstration, early ring QCLs faced several challenges. The feedback strength from the DFB grating was inadequate to sustain stable single-mode emission, causing the excited optical mode to deviate from the intended DFB mode. Consequently, the farfield patterns showed variations, ranging from double-lobed to broad single-lobed shapes, depending on the grating period.

Following these initial developments, the first single-mode ring QCLs were demonstrated shortly thereafter [42]. These devices achieved a beam profile with a full width at half maximum (FWHM) of around 3°, in good agreement with theoretical predictions for this type of laser cavity with a radius of 200 µm. However, the farfield pattern did not display the characteristic interference fringes expected from such a laser. The absence of these fringes was attributed to the presence of the cryostat window, which affected the emitted beam.

This challenge was subsequently addressed with the demonstration of the first ring QCLs capable of room-temperature operation, which marked a significant milestone in the advancement of these devices [40]. A peak optical output power of 120 mW was achieved in pulsed mode, with a reduced threshold current density of approximately 2 kA/cm^2^, which was notably lower than that of comparable Fabry–Pérot (FP) lasers fabricated from the same material, as can be seen in Figure 2(b). These room-temperature ring QCLs operated at an emission wavelength around 8 µm and demonstrated single-mode emission with a side-mode suppression ratio (SMSR) of 30 dB, indicative of strong mode discrimination [40]. Laser spectra of this device are given in Figure 2(c). This improvement enabled the farfield beam to be recorded outside of the cryostat, providing the first clear observation of interference fringes in the farfield pattern of a ring QCL. Such a farfield is depicted in Figure 2(d). The lower threshold current density observed in the ring QCLs compared to the FP lasers is primarily due to the absence of cleaved facets and the corresponding facet-related optical losses. Instead of relying on facet reflectivity, ring QCLs employ a grating for outcoupling the light, resulting in grating-related losses. These grating losses can be more precisely tailored than facet losses, allowing for optimization of the laser performance. This reduction in threshold current density demonstrates the advantage of using a ring geometry to mitigate losses that are more difficult to control in traditional ridge-type or FP QCLs.

A second-order DFB grating deflects light within the laser cavity by 90°, resulting in emission both upwards and downwards. The characteristics of the DFB grating play a crucial role not only in determining the overall optical losses, which dictate the amount of light coupled out of the cavity, but also in influencing the distribution of light emitted in the upward and downward directions. A detailed study conducted by Schwarzer et al. demonstrated how the grating duty cycle (GDC) affects this emission distribution [41]. Figure 2(e) illustrates the different realized GDCs. Specifically, it was found that a GDC of 56 % optimizes the output power directed towards the substrate, while a GDC of 72 % yields the highest output power for surface emission. As summarized in Figure 2(f), the study showed that, when accounting for substrate absorption and reflections, the light refracted toward the substrate is stronger for all GDCs than that directed towards the surface.

### 3.1 Continuous-wave operation

The early ring QCLs were limited to continuous-wave (CW) operation only at cryogenic temperatures [40], which constrained their applicability. For many applications, such as spectroscopy, a CW laser capable of operating at room temperature is highly desirable [43]. The primary challenge in achieving room-temperature CW operation is efficient heat dissipation. In the initial designs, ring QCLs were fabricated in a manner that caused them to protrude from the chip, with both the top surface, where the grating is located, and the sidewalls exposed to the surrounding environment. Although a SiN isolation layer and a gold contact layer protect the laser cavity, these layers are thin and do not provide sufficient heat dissipation, and the surrounding air is a poor thermal conductor.

**Figure 3:**
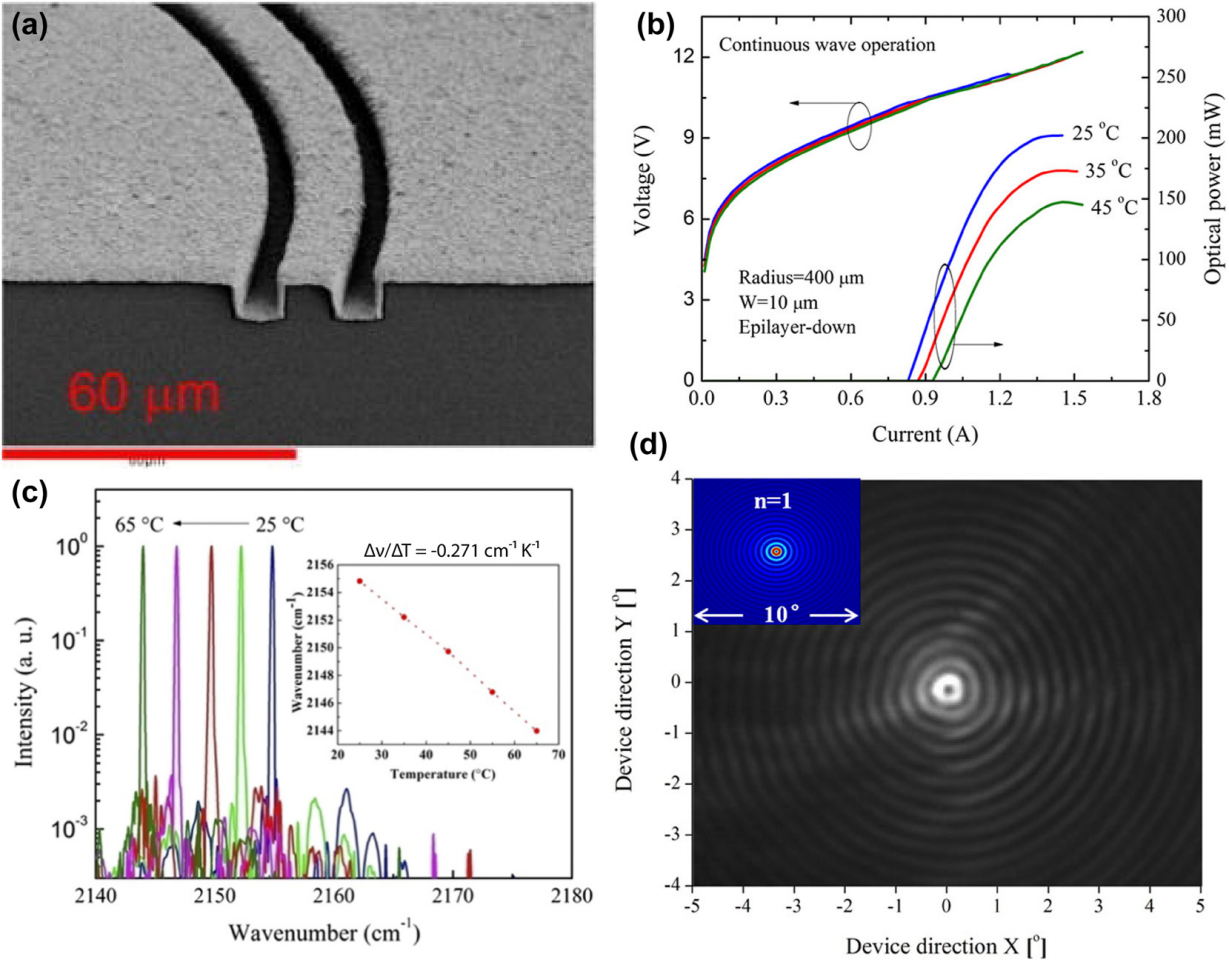
Room-temperature CW operation of ring QCLs. (a) SEM image of a CW ring QCL overgrown with InP and electroplated with 3 µm gold. (b) LIV characteristics of a CW ring QCL at room temperature with a maximum output power of 202 mW. The threshold current of 0.9 A corresponds to a threshold current density of 3.6 kA/cm^2^. (c) Single-mode spectra with an SMSR of 25 dB and temperature-tuning behavior with a tuning coefficient of −0.271 cm^−1^/K. (d) Recorded farfield pattern of a CW ring QCL at room temperature. The inset depicts the simulated farfield. (a) Is reprinted from Ref. [44], with permission from AIP Publishing; (b–d) are reprinted from Ref. [45], with permission (CC BY 4.0).

A solution to this thermal management problem was demonstrated in 2011 by Bai et al., who introduced an overgrowth of the ring QCL with InP [44]. An SEM image of this overgrown ring QCL is provided in Figure 3(a). They chose a ring radius of 400 µm and a buried second order DFB grating was fabricated from an InGaAs layer, located directly on top of the active region. This approach enabled significant improvements in laser performance, achieving a maximum output power of 510 mW, a threshold current density of 1.06 kA/cm^2^, a wall-plug efficiency of 6.25 %, and a slope efficiency of 1.08 W/A, all under CW operation at room temperature. However, the overgrown ring QCL did not operate at the intended DFB mode, as evidenced by a broad farfield pattern that indicated the device was operating in a higher-order mode. This overcoupling was attributed to the close proximity of the DFB grating to the active region, which resulted in excessive feedback.

To address these issues, Wu et al. refined the design by increasing the distance between the grating and the active region through the introduction of a surface grating [45]. This modification was complemented by efficient heat dissipation achieved via electroplating of a 2 µm thick gold layer, followed by epilayer-down bonding of individual devices onto an AlN submount. This improved processing approach allowed for single-mode emission at the correct DFB mode and produced the expected farfield pattern with clearly defined interference fringes. However, these improvements came at the cost of reduced performance. The epilayer-down bonded ring QCL exhibited a lower output power of 202 mW (see Figure 3(b)), a higher threshold current density of 3.3 kA/cm^2^ and reduced wall-plug efficiency and slope efficiency of 1.32 %, and 0.57 W/A, respectively, compared to the overgrown device. Figure 3(c) depicts the spectra of this CW ring QCL. The farfield pattern shown in Figure 3(d) distinctly displays circular interference fringes, characteristic of the laser’s operation at the DFB mode.

### 3.2 Microrings

**Figure 4:**
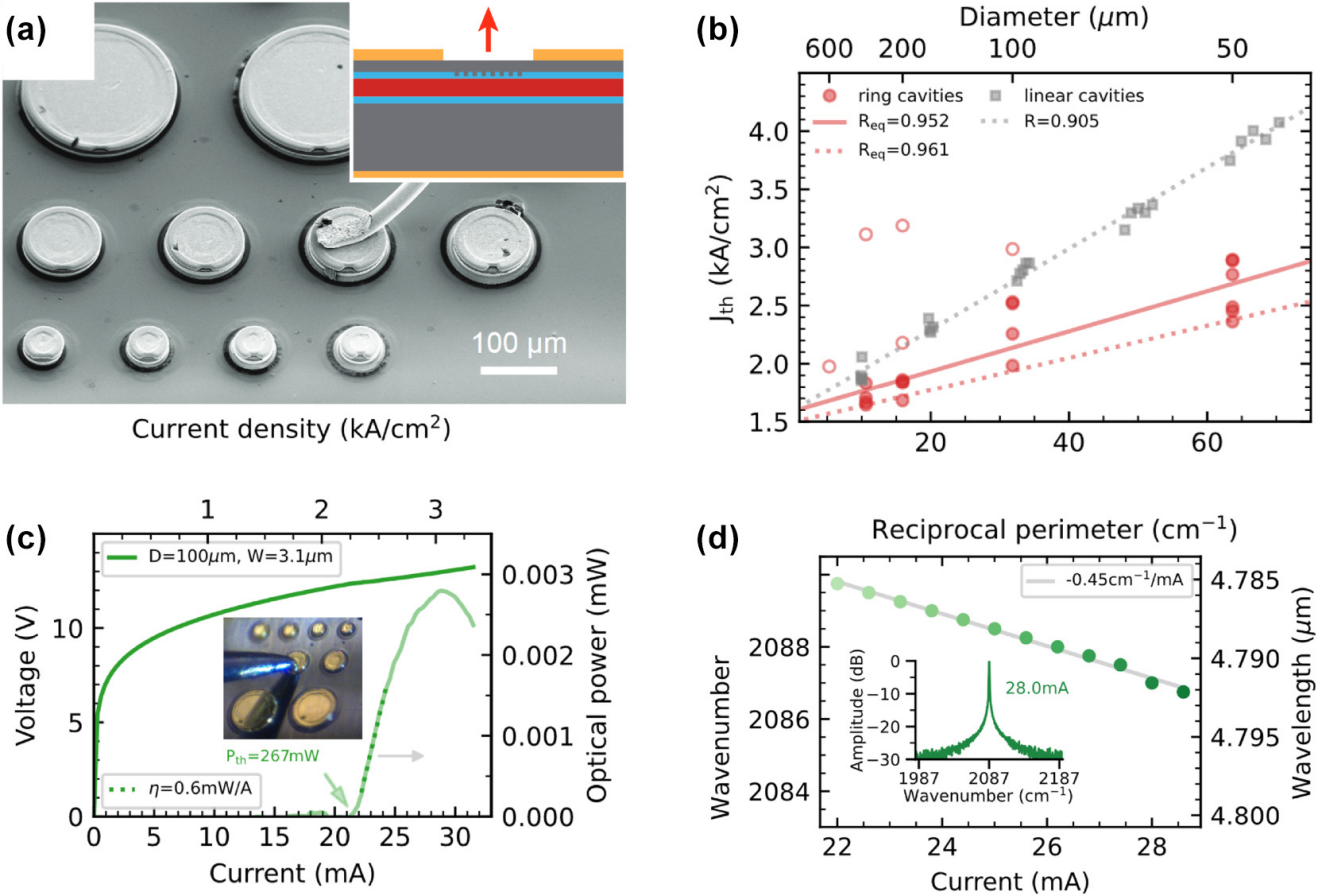
Miniaturization and performance of microring QCLs. (a) SEM image of microring QCLs with different radii. The inset shows a schematic of the cross section of the device with the location of the buried DFB grating indicated in blue and the active region indicated in red. (b) Threshold current density as a function of the microring radius. The smallest lasing microring exhibits a radius of 50 µm. (c) LIV characteristics of a CW microring QCL. The inset shows a photograph during on-chip testing with a needle probe. (d) CW current-tuning characteristics and single-mode spectrum (inset). (a–d) Are reprinted from Ref. [46], with permission (CC BY 4.0).

Reducing the footprint of surface-emitting QCLs is critical for enabling large-scale production of low-cost, low-power MIR lasers. Achieving this requires a low-loss outer boundary to support the whispering gallery mode (WGM), which confines light within the microring structure. Stark et al. realized such boundary optimization by employing an Al_2_O_3_/Au coating along the outer cavity boundary for microrings of different sizes [46]. Ring QCLs with different diameters are given in Figure 4(a).

This configuration leverages an overgrowth approach with a buried heterostructure and a short arcuate grating section, etched directly into an InGaAs layer atop the active region, a method comparable to that of Bai et al. [44]. Stark et al. provide an in-depth analysis showing that the threshold current density increases as the ring diameter decreases, demonstrating lasing even in rings as small as 50 µm, as shown in Figure 4(b). Moreover, they achieved CW operation in rings with diameters as small as 100 µm. These CW microrings exhibit relatively low power dissipation, with values of 267 mW at threshold and 374 mW at maximum output. The corresponding LIV curve is presented in Figure 4(c). The slope efficiency is reported at 0.6 mW/A, and single-mode emission at around 4.8 µm shows an SMSR of 25 dB, with a current-tuning coefficient of −0.45 cm^−1^/mA. An exemplary spectrum as well as the current-tuning behavior in CW mode are illustrated in Figure 4(d). This study marks a significant step toward the scalable, low-cost fabrication of surface-emitting QCLs with minimized power consumption, though farfield pattern data for these microring QCLs was not available at the time of writing this review.

### 3.3 Terahertz emission

THz radiation, occupying the frequency range between infrared and microwave, is increasingly important due to its unique applications in fields such as spectroscopy, imaging, and communication. THz waves can penetrate non-metallic materials and provide high-resolution imaging without the ionizing effects of X-rays. However, generating coherent THz radiation, crucial for many of these applications, remains a challenge. A coherent emitter, such as a QCL, provides the narrow linewidth and phase coherence necessary for precision in spectroscopy and communication systems [47].

In 2008, Mahler et al. demonstrated a quantum cascade microdisk laser operating in the THz region, featuring a second-order DFB grating for vertical light outcoupling [48]. This device emitted at 3.3 THz and had a radius around 90 µm, showcasing a key step forward for surface-emitting THz QCLs. Typically, THz QCLs are fabricated using a double-metal waveguide, where the gain material is sandwiched directly between two metal layers. This design enables sub-wavelength confinement of the optical mode, resulting in a confinement factor close to unity, but also causes the emitted beam to be highly divergent when exiting through a standard FP laser facet. Therefore, a circular surface-emitting geometry based on second-order DFB gratings, which significantly increases the emitting area, is even more advantageous in the THz range than in the MIR, where similar strategies have been employed.

**Figure 5:**
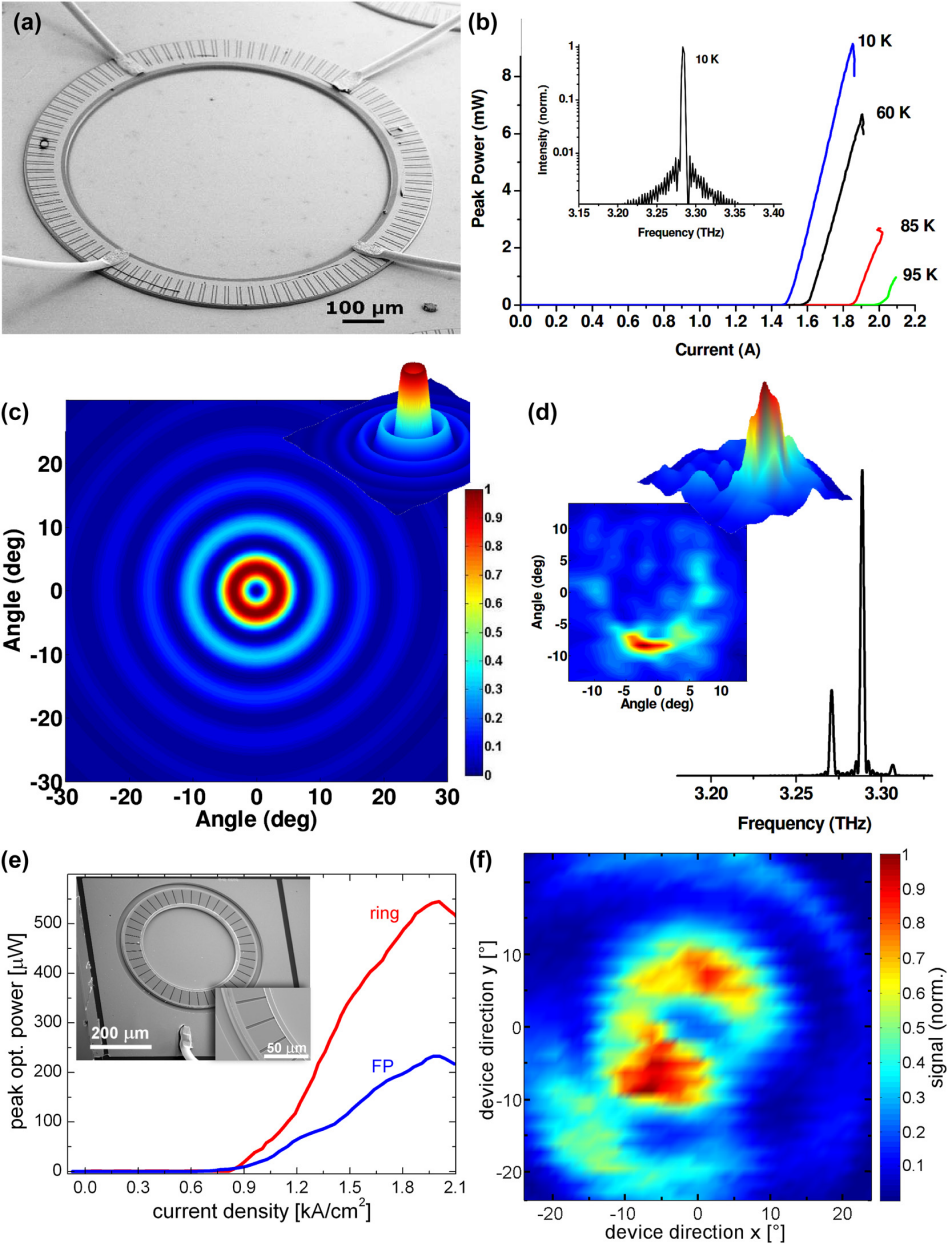
Ring QCLs operating in the THz regime. (a) SEM image of a THz ring QCL with a double-slit DFB grating. (b) LIV characteristics at different temperatures. The threshold currents of 1.6 A at 60 K and 2.0 A at 95 K correspond to threshold current densities of 0.59 kA/cm^2^ and 0.74 kA/cm^2^, respectively. The inset shows a spectrum with an SMSR of 20 dB. (c) Simulated farfield pattern. (d) Recorded farfield and spectrum of the THz ring QCL in the double-slit configuration. (e) LIV characteristics of a THz ring QCL in the narrow-slit configuration. The optical output power is larger than in a comparable FP device. The inset shows an SEM image of the ring QCL. (f) Recorded farfield of a THz ring QCL with rotational symmetry and interference rings. (a–d) Are reprinted from Ref. [49], under the terms of the Open Access Publishing Agreement. ©2009 Optical Society of America; (e–f) are reprinted from Ref. [50], with permission from AIP Publishing.

In 2009, two separate groups independently demonstrated ring QCLs in the THz regime [49], [50]. Mahler et al. introduced a ring QCL with a radius of around 500 µm and a double-slit configuration for the DFB grating [49], aimed at providing a more uniform current injection. An SEM image of the double-slit grating configuration is given in Figure 5(a). This design positioned a gold pumping segment at the center of each grating slit, slightly smaller than the slit itself, creating the double-slit configuration. The device achieved a maximum optical output power of 10 mW, a slope efficiency of 25 mW/A, a threshold current of 1.5 A, and operated up to a temperature of 100 K, as shown in Figure 5(b). However, the farfield pattern was not uniform, indicating non-ideal emission characteristics. Figure 5(c) and (d) show the simulated and recorded farfield patterns of a THz ring QCL featuring the double-slit configuration, respectively.

Mujagic et al. took a different approach using smaller rings with a radius of 200 µm. Furthermore, they avoided the double-slit configuration and instead used single, narrower slits compared to those used in MIR QCLs [50]. This significantly smaller design, while producing a lower optical output power of 0.55 mW and a slope efficiency of 0.97 mW/A, achieved a higher maximum operating temperature of 120 K and a lower threshold current of 0.52 A. The LIV as well as an SEM image of this THz ring QCL is given in Figure 5(e). More importantly, the farfield pattern exhibited the expected circular symmetry, as can be seen in Figure 5(f). The clear interference fringes indicate a uniform and coherent emission from the entire ring surface. Additionally, the THz ring QCL demonstrated an optical output power and slope efficiency that were twice those of an FP device fabricated from the same material, further highlighting the advantages of the surface-emitting circular geometry for THz applications.

### 3.4 Spectral tuning and array integration

One key application of ring QCLs is spectroscopy, where a detailed understanding of the laser’s tuning characteristics is crucial. In a comprehensive study, Brandstetter et al. analyzed the time-resolved spectral behavior of pulsed ring QCLs and compared it to that of straight DFB FP devices [51]. Utilizing a high-resolution step-scan Fourier transform infrared (FTIR) method, they achieved a spectral resolution of 0.1 cm^−1^ and a time resolution of 2 ns. The study compared a short DFB FP laser, a long DFB FP laser, and a ring QCL, all fabricated from the same material. The short DFB FP laser and the ring QCL both had a similar length of 0.9 mm, where the length of the ring corresponds to its circumference. The investigation focused on the stability of spectral emission, which depends on the cavity length L and the coupling coefficient *κ* through the coupling strength factor *κ*⋅L [52]. Based on this relationship, the short DFB FP laser was expected to exhibit similar performance to the ring QCL.

**Figure 6:**
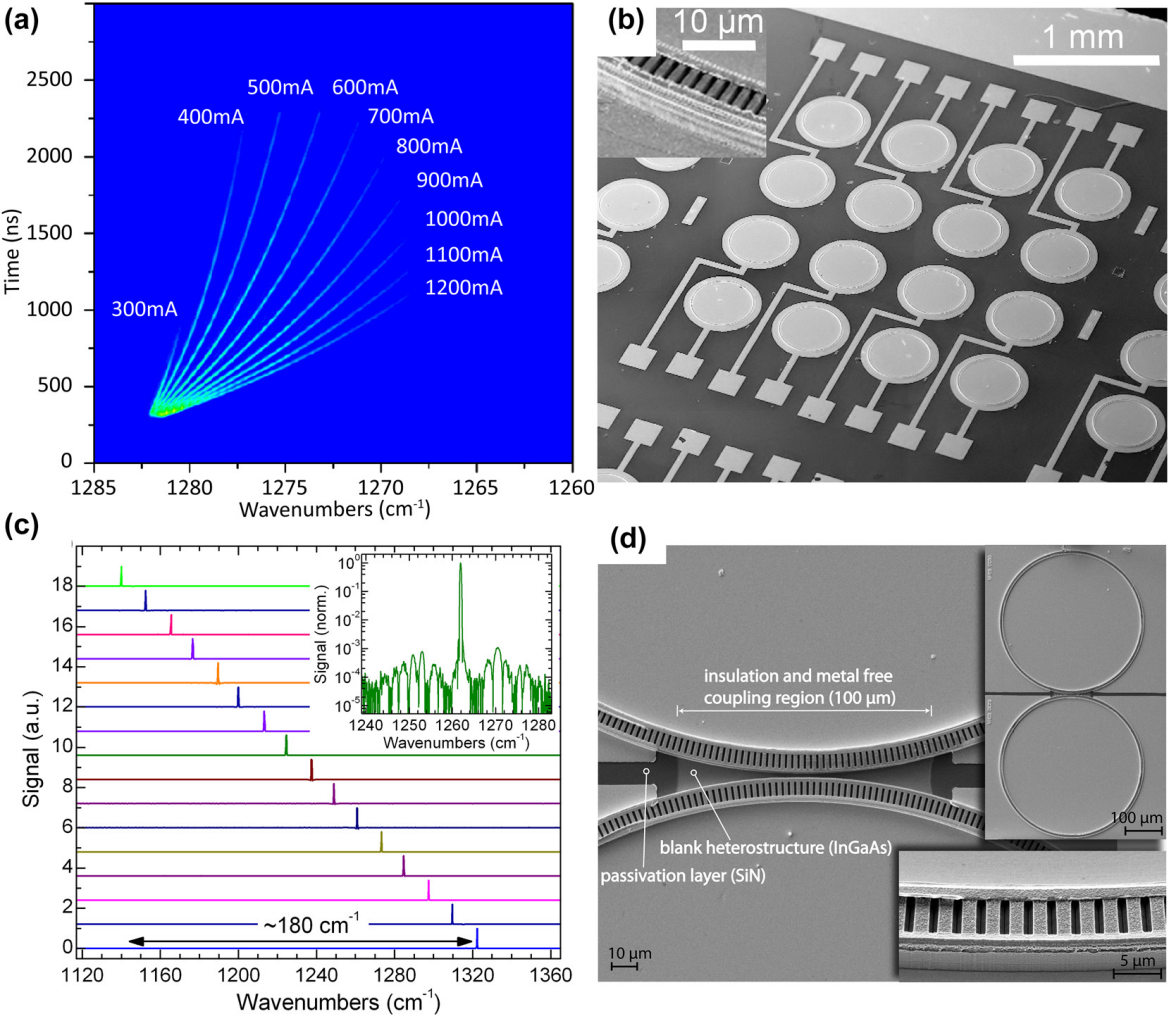
Spectral tuning and array integration of ring QCLs. (a) Intra-pulse tuning characteristics for different currents with a maximum tuning range of 12.9 cm^−1^ of a single ring QCL. (b) SEM image of a surface-emitting ring QCL array with 16 lasers at different wavelengths. (c) Emission spectra of the 16 ring QCLs in the array. All devices cover a spectral range of 180 cm^−1^. The inset shows one of those spectra with an SMSR of 30 dB. (d) SEM images of coupled rings. (a) Is reprinted from Ref. [51], under the terms of the Open Access Publishing Agreement. ©2014 Optical Society of America; (b–c) are reprinted from Ref. [53], with permission from AIP Publishing; (d) is reprinted from Ref. [54], with permission from AIP Publishing.

Surprisingly, the results revealed that the ring QCL exhibited a threshold current density comparable to that of the long DFB FP laser. Moreover, the stable emission range of the ring QCL extended over an even broader spectral range than the long DFB FP laser. This performance is attributed to the absence of facets in the ring QCL, highlighting the advantages of facetless laser designs in terms of mode stability and extended spectral coverage. Figure 6(a) gives a summary of the spectral tuning characteristics of ring QCLs.

Array integration of ring QCLs is a promising approach for applications in gas analysis and chemical sensing, where multiple single-mode laser sources are essential for measuring various substances. Mujagic et al. demonstrated the first ring QCL array, consisting of 16 ring QCLs, achieving a total tuning range of 180 cm^−1^ around a center wavelength of 8.2 µm [53], [55]. Figure 6(b) depicts an SEM image of the 16 ring QCL array and Figure 6(c) outlines the spectrum of each of those 16 devices. The lasers function in single-mode operation, corresponding to their specific grating period, and exhibit a consistent output without mode hops. The facetless design of ring QCLs makes them particularly appealing for use in laser arrays, as their performance is governed by the gain material utilized rather than being influenced by the frequency selection process. Such monolithically integrated arrays facilitate compact, widely tunable light sources, thereby enhancing selectivity and sensitivity for multianalyte spectroscopy. Two-dimensional integration of these coherent emitters allows for on-wafer testing and scalability, thus reducing fabrication costs and efforts. This advancement in array integration marks a significant step toward the development of sophisticated spectroscopic tools leveraging the unique capabilities of ring QCLs.

The linear arrangement of ring QCL arrays poses certain limitations, particularly in terms of space consumption on the semiconductor chip, which restricts the number of devices that can be integrated. To address this, alternative designs incorporate two or more concentric rings that share the same optical axis [37], [56]. These configurations optimize chip area utilization, enabling higher device density and potentially more compact laser arrays without compromising functionality.

### 3.5 Coupled rings

The modes within ring resonators are known as WGMs, which are concentrated near the outer wall of the ring. This results in a significant amount of mode leakage at the ring’s outer border, manifesting as an evanescent field. This field can facilitate coupling between rings through evanescent field interactions. In a study by Schwarzer et al., coherent coupling and phase locking of two ring QCLs were successfully demonstrated [54], [57]. An SEM image of two coupled ring QCLs is shown in Figure 6(d). This achievement highlights the potential of ring QCLs for applications in coherent, high-power surface-emitting QCL arrays.

In their study, two rings with a coupling gap of 1 µm demonstrated clear coupling effects. This was evident in the reduced threshold current density observed when both rings were operated together, compared to when a single ring was active. The elevated threshold current density for single-ring operation is attributed to additional losses from mode leakage. However, when both rings are active, the leaking modes are able to couple into the adjacent ring, preventing these losses and suggesting effective coupling through evanescent fields between the rings. Consequently, this coupling effect reduces the threshold current density during simultaneous operation of both rings.

Additionally, the study demonstrated synchronization in the form of injection locking between the two rings. When operated separately, the two identical rings exhibited identical emission spectra. By introducing an offset current to one of the rings, the spectral peaks shifted, showing detuning. However, when the rings were operated simultaneously (one with normal current and the other with an offset current), a single spectral peak was observed, demonstrating that the optical modes synchronized. In contrast, when the coupling gap was increased to 3 µm, the two individual spectral peaks persisted even when both rings were operated simultaneously, indicating that no mode locking occurred at this larger separation.

In addition to coupling between ring QCLs, coupling a ring QCL to a linear waveguide has emerged as an important concept, particularly in applications involving frequency combs and soliton generation. In such designs, the ring resonator does not employ a second-order DFB grating for surface emission. Instead, the mode of the ring is evanescently outcoupled or couples into a linear waveguide positioned in close proximity, which guides the light to its facet for emission. The absence of the grating prevents the formation of standing waves within the ring, enabling a traveling wave regime. However, it has been demonstrated that a small but finite amount of backscattering can help stabilize soliton solutions by acting as a phase reference [58]. This mode control is essential for soliton formation, paving the way for new approaches in nonlinear optics and advancing MIR spectroscopy through compact frequency comb sources [59], [60], [61], [62], [63], [64].

## 4 Emission beam

### 4.1 Grating-induced beam modifications

One of the most notable advantages of ring QCLs is their capability to produce a circularly symmetric and collimated emission beam. Due to the ring-shaped cavity, the farfield pattern resembles the two-dimensional equivalent of a double-slit diffraction pattern, as indicated in Figure 1(d). This results in concentric interference rings in the farfield. Similar to the double-slit experiment, the farfield characteristics of a ring QCL are governed by two main factors. First, the ring’s diameter, for a given wavelength, influences the distance between the concentric interference rings, analogous to the slit separation in the double-slit experiment. Second, the width of the waveguide, specifically the width of the emitting region, determines the envelope of the farfield intensity profile, akin to the slit width in a double-slit experiment.

However, unlike the conventional double-slit experiment, ring QCLs exhibit a unique polarization behavior due to their inherent TM polarization. When employing conventional radial grating slits, the resulting emission is tangentially polarized. Since the entire ring is coherent and in-phase, opposite sides of the ring have antiparallel polarization vectors. In the farfield, these opposing components interfere destructively, resulting in a central intensity minimum. This is analogous to a double-slit experiment in which one slit is covered by a plate with a thickness of *λ*/(2 (*n*−1)), where *λ* is the wavelength and *n* is the refractive index of the plate.

**Figure 7:**
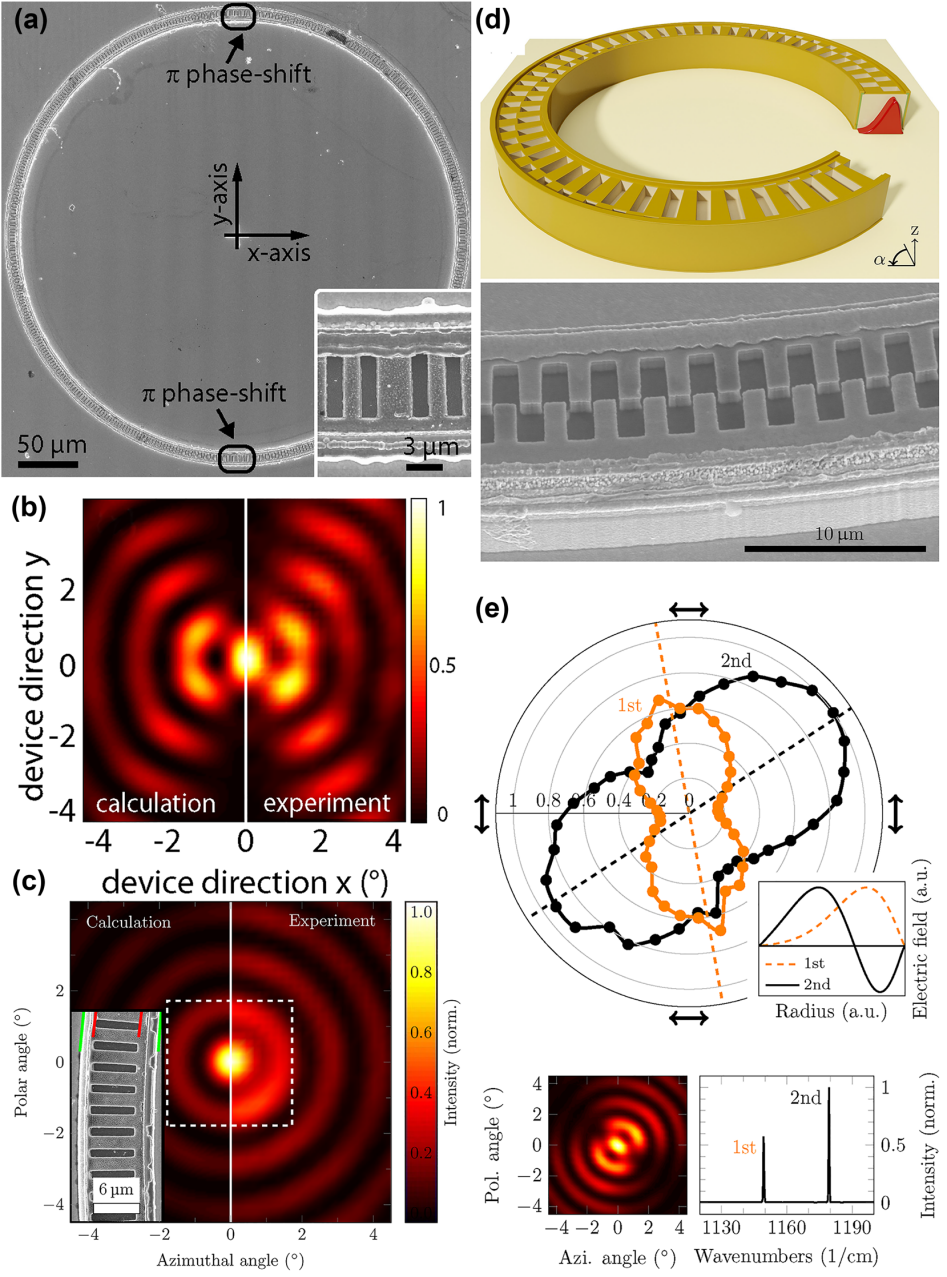
Grating engineering and polarization control in ring QCLs. (a) SEM image of a ring QCL with two *π* phase shifts in the DFB grating. The inset shows a close-up image of the phase shift. (b) Farfield pattern of a ring QCL with two *π* phase shifts. The central intensity maximum is linearly polarized. (c) Farfield pattern of a ring QCL with an off-center grating (inset). The central intensity lobe contains all polarizations. (d) Sketch (top) and SEM image (bottom) of a ring QCL with a dual *π* phase shifted grating. (e) Results of the polarization-dependent spectral measurements of a ring QCL with a dual grating (top). The two distinct emission wavelengths (bottom, right) exhibit opposite polarization dependencies, which indicates a first-order and second-order WGM and explains the rotation of the farfield (bottom, left). (a–b) Are reprinted from Ref. [39], with permission from AIP Publishing; (c) is reprinted from Ref. [65], under the terms of the Open Access Publishing Agreement. ©2014 Optical Society of America; (d–e) are reprinted from Ref. [66], with permission (CC BY 4.0).

While a well-collimated emission beam is desirable, the presence of a central minimum in the intensity profile can be problematic for some applications. To address this, Schwarzer et al. demonstrated ring QCLs featuring a second-order DFB grating with two *π* phase shifts on opposite sides of the ring [39], as illustrated in the SEM image in Figure 7(a). This design rotates the polarization vector by 180° over half of the ring, leading to constructive interference at the center of the farfield pattern and creating a central intensity maximum, as observed in Figure 7(b). The resulting central lobe is linearly polarized, while sharp intensity minima appear at the phase shift locations due to localized destructive interference.

Babichev et al. explored an alternative method by introducing grating phase shifts using focused ion beam (FIB) milling [67], [68]. However, these gratings did not achieve single-mode operation at the desired grating mode. In a different approach, they utilized a partial distributed Bragg reflector (DBR) grating, covering only a section of the ring, to produce farfield patterns similar to those seen in straight surface-emitting DFB lasers [69], [70].

A different innovative strategy was demonstrated by Szedlak et al., who implemented an off-center grating design [65]. This approach introduces an effective chirp to the grating, creating a continuous *π* phase shift rather than an abrupt one. The continuous phase shift enables phase-matched wavefronts, resulting in constructive interference and a central intensity maximum. Unlike abrupt phase shifts, which create dark spots in the near- and farfield patterns, this approach yields a smooth intensity distribution around the ring waveguide, as can be seen in Figure 7(c). Additionally, while abrupt phase shifts produce a linearly polarized central lobe, off-center gratings generate a central lobe containing all polarizations.

Another notable grating modification involves rotating the grating slits away from the radial direction. By employing non-radial slits, the emitted polarization can be rotated by the same angle as the grating slits, effectively allowing the grating to function as a polarizer [65]. This versatility in tailoring the polarization characteristics provides ring QCLs with additional degrees of freedom for applications where polarization control is crucial.

Grating-induced beam modifications not only allow for improving the farfield characteristics of ring QCLs, such as achieving a central intensity maximum through *π* phase shifts or off-center gratings, but also offer a valuable tool for examining the internal mode properties of these devices. As mentioned earlier, ring QCLs support WGMs, which are characterized by their confinement near the outer wall of the waveguide. The degree of this outward shift is determined by the curvature of the ring, with more pronounced shifts occurring in rings with smaller radii. Comprehensive investigations of WGM profiles have been previously conducted using nearfield probes [71], photon scanning tunneling microscopy [72], and thermocouple probes [73].

For the first time, Szedlak et al. introduced a novel technique to map WGMs in ring QCLs using farfield emission characteristics [66]. This technique employs a dual DFB grating, consisting of two parallel gratings that are phase shifted by *π* relative to each other. Along the circumference of the ring, the ratio between these two gratings is varied continuously to induce a gradual phase shift. A sketch as well as an SEM image of this dual grating are depicted in Figure 7(d). Since the two gratings are *π* phase shifted, light emitted from these gratings experiences destructive interference, and the location of maximum destructive interference depends on the spatial overlap between the WGMs and the grating structures.

In a hypothetical scenario, where the WGM would be symmetrically centered within the waveguide, the point of maximum destructive interference would coincide with the segment of the ring where the gratings are balanced with a 50:50 ratio. However, due to the outward displacement of the WGM, this position shifts, causing a corresponding rotation of the near- and farfield intensity patterns. By measuring this rotation, the center of mass (COM) of the WGM can be determined with precision.

The study predominantly found an outward shift of the COM, which confirmed that most ring QCLs exhibit first-order WGMs, validating the theoretical expectations. However, an unexpected observation was made for certain devices: a farfield rotation in the opposite direction, indicating an inward shift of the mode’s COM and suggesting a higher order mode. Spectral analysis of these devices revealed the presence of two distinct spectral peaks, indicating that the laser was supporting multiple modes. A more in-depth polarization-dependent study of the farfield rotation identified these modes as first-order and second-order WGMs, as outlined in Figure 7(e). This highlighted the effectiveness of this mapping technique in discerning complex modal behaviors within the ring QCL cavity, offering insights that would be challenging to obtain using conventional methods. The ability to precisely characterize and control WGM profiles in ring QCLs is not only valuable for understanding device physics but also critical for optimizing laser performance and improving beam quality.

### 4.2 On-chip optical elements

One of the significant advantages of ring QCLs lies in their vertical emission capability, where light is radiated both upwards into the air and downwards through the substrate. This dual-directional emission, coupled with a large emission area, naturally produces collimated beam profiles. Notably, the downward emission through the substrate allows for on-chip modifications of the emission beam, opening up new possibilities in beam control and manipulation.

In a pioneering approach, Schwarzer et al. integrated a *π* shift ring QCL with a gold wire grid deposited on the back side of the substrate after the ring QCL was fabricated [39]. The wire grid functioned as an on-chip polarizer, delivering polarization properties comparable to external polarizers. This on-chip polarizer not only extended the polarization characteristics of the central lobe to the entire farfield but also demonstrated the feasibility of substrate-based modifications in ring QCLs.

**Figure 8:**
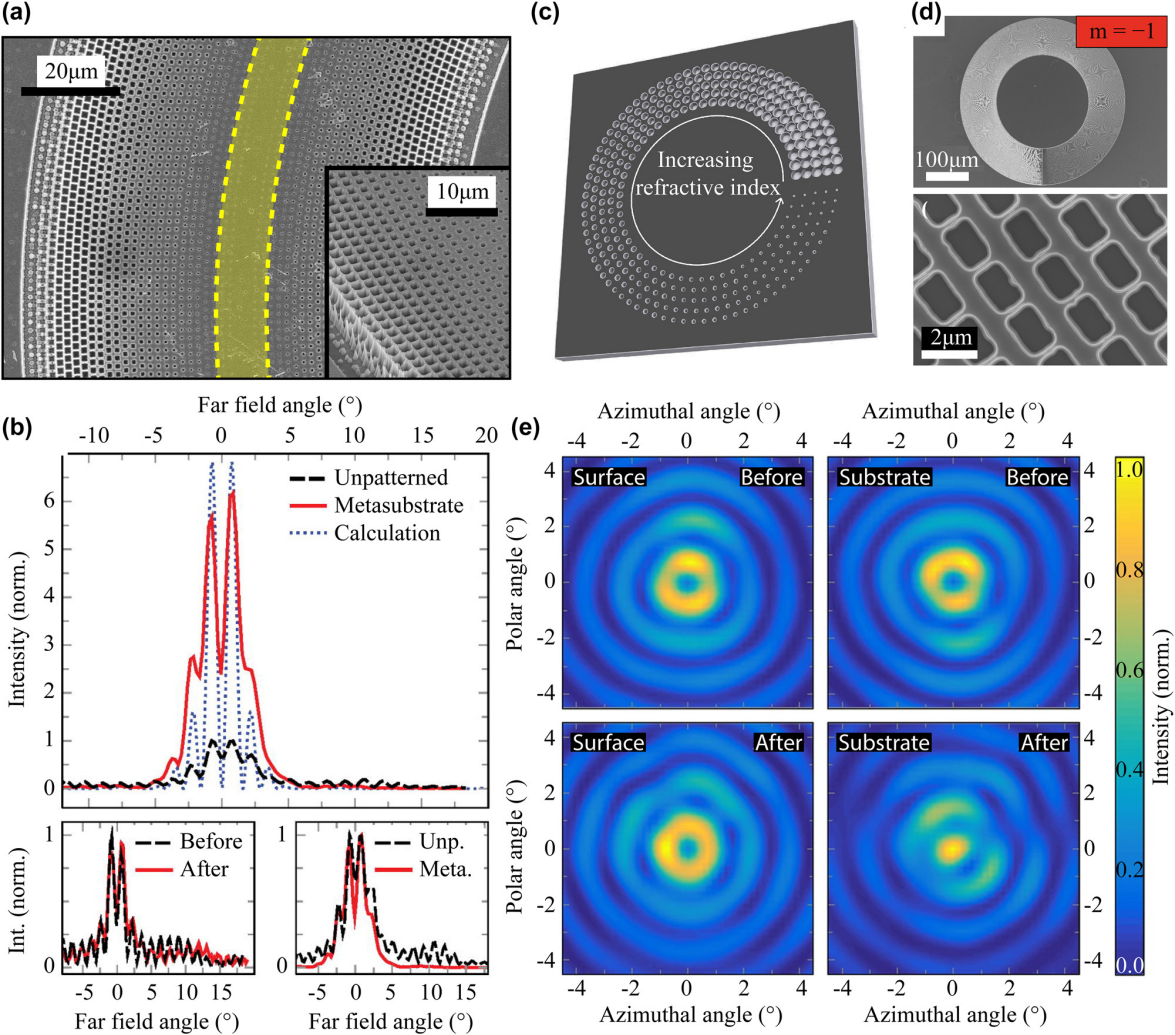
Integrated photonic elements for beam shaping in ring QCLs. (a) SEM images of the metasubstrate. The yellow stripe indicates the location of the ring QCL on the other side of the semiconductor chip. (b) Farfield pattern with and without the metasubstrate. The top panel shows the farfield patterns of the substrate-emitted beam, scaled to the peak intensity prior to the fabrication of the metasubstrate. This demonstrates the more than six-fold peak intensity increase. The bottom left panel shows the surface-emitted beam without any notable difference, indicating that the overall performance of the laser did not change. The bottom right panel shows the data from the top panel but both farfields are now scaled to unity, which indicates that the metasubstrate collects intensity from larger emission angles and shifts it towards the beam center. (c) Sketch of an OAM metasubstrate. (d) SEM images of an OAM metasubstrate with topological charge *m* = −1. (e) Comparison of the farfields before and after fabrication of the OAM metasubstrate. While the surface-emitted farfield does not show a notable change, the substrate-emitted beam switches from central minimum to maximum, indicating a beam carrying OAM. (a–b) Are reprinted from Ref. [74], with permission from AIP Publishing; (c–e) are reprinted from Ref. [75], with permission (CC BY 4.0).

Expanding on this concept, Szedlak et al. introduced an innovative metasubstrate, an on-chip lens for beam collimation, fabricated directly on the back side of the ring QCL’s substrate [74]. This metasubstrate, as outlined in Figure 8(a), operates as a gradient index element, designed to phase-match rays emitted at different angles from the ring laser by introducing distinct optical path lengths. The gradient in the refractive index is achieved by etching sub-wavelength holes with varying diameters into the back side of the substrate, a process carefully controlled through aspect-ratio-dependent etching. The total etch depth poses a limitation on the overall size of the metasubstrate, which ultimately influences the lens area and performance.

The effectiveness of this metasubstrate design is evident from the farfield patterns. When comparing the farfield before and after the implementation of the metasubstrate on the same ring QCL, a more than six-fold increase in the central intensity maximum was observed, without affecting the total output power. The corresponding farfield patterns are provided in Figure 8(b). This substantial increase confirms the metasubstrate’s function of redirecting intensity from larger emission angles towards the central lobe. The combination of a ring QCL with grating *π* shifts and a metasubstrate of equivalent size could theoretically yield a seven-fold enhancement in the central intensity maximum [76].

Beyond simple beam collimation, this gradient index approach opens up avenues for diverse beam-shaping applications. For example, Figueiredo et al. proposed an on-chip solution based on an azimuthal refractive index gradient, fabricated through grayscale lithography, aimed at generating a beam profile with a central intensity maximum [77]. In a different realization, Szedlak et al. employed sub-wavelength holes to create an azimuthal refractive index gradient, which introduces a phase shift along the azimuthal direction, twisting the emitted wavefront and producing orbital angular momentum (OAM) beams [75]. These OAM metamaterials are shown in Figure 8(c) and (d). They influence the farfield pattern significantly and can be used to demonstrate various topological charges.

For instance, a ring QCL with an OAM metasubstrate possessing a topological charge of 1 was fabricated. The corresponding farfields are displayed in Figure 8(e). The left panels show the emission in the surface direction before (top) and after (bottom) fabrication of the metasubstrate. As expected, these farfields remain largely unchanged, both exhibiting a central intensity minimum. The right panels depict emission through the substrate, again before (top) and after (bottom) metasubstrate fabrication. While the unstructured device emits identical beam profiles in both directions, the metasubstrate significantly modifies the substrate-emitted beam by converting the central intensity minimum into a pronounced maximum. This transformation indicates the generation of an OAM beam with a topological charge of 1. In this configuration, a total phase shift of 2*π* is distributed around the entire circumference of the ring, resulting in opposite sides of the ring exhibiting a *π* phase shift relative to each other. This structure produces a central intensity maximum and maintains rotational symmetry in the farfield, confirmed for both clockwise and counter-clockwise OAM beams. Additionally, a ring QCL with a topological charge of 2 was also demonstrated, showing a characteristic central intensity minimum, consistent with theoretical predictions based on independent dipole models [78] and vectorial ray-based diffraction integrals [79].

This research underscores the extraordinary flexibility of surface-emitting ring QCLs, where precise modifications of the emission beam can be achieved through the substrate. Such beam engineering would be considerably more challenging in traditional facet-emitting lasers, highlighting the unique advantages of the ring QCL architecture for advanced photonic applications.

## 5 Spectroscopic applications

QCLs cover an extensive spectral range, from 2.6 µm to 250 µm [80], making them highly suitable for probing rotational and vibrational resonances in numerous molecules. Their broad tunability and narrowband, mode-hop-free single-mode operation make them ideal for spectroscopic applications, especially in the MIR region with its plethora of molecular fingerprints. Ring QCLs, with their surface-emitting design, provide a unique combination of stable single-mode output, circularly symmetric beam profiles, and the potential for wide spectral tuning.

**Figure 9:**
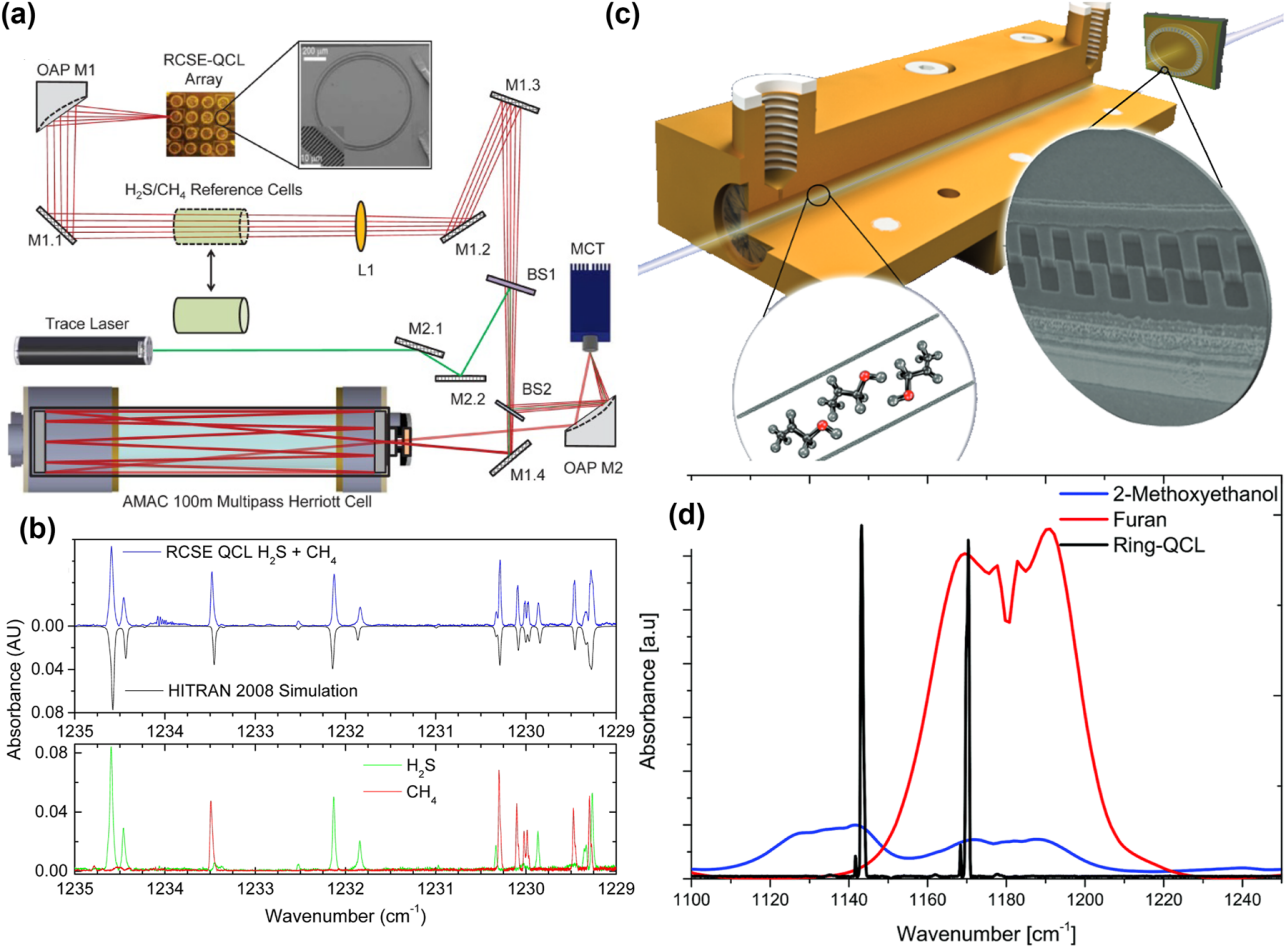
Ring QCLs in advanced gas detection and molecular spectroscopy. (a) Sketch of an experimental setup featuring a ring QCL and a Herriott cell. (b) Recorded spectra of H_2_S and CH_4_. (c) Sketch of dual-color ring QCL, incorporating a dual grating structure, with an iHWG. (d) Emission spectrum of the dual-color ring QCL with the absorption spectra of the two analytes under test. (a–b) Are reprinted from Ref. [81], under the terms of the Open Access Publishing Agreement. ©2016 Optical Society of America; (c–d) are reproduced from Ref. [82] with permission from the Royal Society of Chemistry (CC BY 3.0).

Moser et al. demonstrated, for the first time, the potential of ring QCLs in high-sensitivity gas sensing by employing a 100 m Herriott cell to achieve a detection limit of 1.5 ppmv for H_2_S in N_2_ [81]. In their setup (see Figure 9(a)), 300 ns pulses enabled a spectral tuning over 1.5 cm^−1^ around 8.1 µm. By extending the pulse duration to 3 µs, they achieved a mode-hop-free tuning range of 8.5 cm^−1^, enabling the simultaneous targeting of multiple absorption lines of H_2_S and CH_4_, as demonstrated in Figure 9(b). This spectral agility, combined with low-pressure operation, allowed for selective and interference-free detection of H_2_S in a methane matrix.

Building on this success, ring QCLs have found additional applications in advanced spectroscopic systems. As represented in Figure 9(c), Tütüncü et al. implemented a novel combination of a ring QCL with a substrate-integrated hollow waveguide (iHWG) [83], which is an emerging solution for portable and miniaturized gas sensing [82]. iHWGs offer substantial advantages over conventional multipass cells, such as compact size, simplified optical alignment, and a significantly reduced sample volume, which accelerates sample exchange times. The iHWG design, with a gas volume of just 600 µL over a length of 7.5 cm, enhances the so-called volumetric optical efficiency. This high efficiency stems from its combination of small sample volume, efficient optical confinement, and superior signal-to-noise ratio.

The ring QCL employed in this work was a dual-color laser device operating near 8.6 µm, utilizing a dual grating structure that supports both first- and second-order WGMs [66]. The current-dependent nature of these WGMs allows for dynamic control over the emission spectrum. At low currents, only one WGM peak is present, but at higher currents, a second peak emerges, enabling the simultaneous detection of different gases in a mixture. Laser and absorption spectra are given in Figure 9(d). In their study, Tütüncü et al. detected and quantified furan and 2-methoxyethanol using a single dual-color ring QCL. The system achieved a coefficient of determination (*R* ^2^) greater than 0.99 over a concentration range of 2–80 % of furan in 2-methoxyethanol.

This novel dual-color ring QCL-based iHWG platform offers a compact and efficient solution for MIR gas sensing applications, particularly in scenarios demanding rapid and accurate measurements of transient gas compositions. The spectral versatility of ring QCLs in combination with miniaturized iHWG technology paves the way for robust and highly sensitive portable sensing systems in the MIR spectral domain.

### 5.1 Modulation characteristics and dispersion spectroscopy

Modulation characteristics play a critical role in the application of ring QCLs for advanced spectroscopic techniques beyond conventional setups that rely on the Beer–Lambert law. Such techniques include wavelength- and frequency-modulation spectroscopy [84], [85], intrapulse absorption spectroscopy [86], chirped laser dispersion spectroscopy [87], and heterodyne phase-sensitive dispersion spectroscopy (HPSDS) [88]. These methods capitalize on the detailed understanding of the laser’s tuning behavior in response to modulation. In HPSDS, the laser current is modulated at radio-frequency (RF) rates to generate sidebands, and the technique measures the refractive index spectrum by probing the phase of the RF-modulated light, making it immune to power fluctuations and reducing the need for calibration [89], [90].

**Figure 10:**
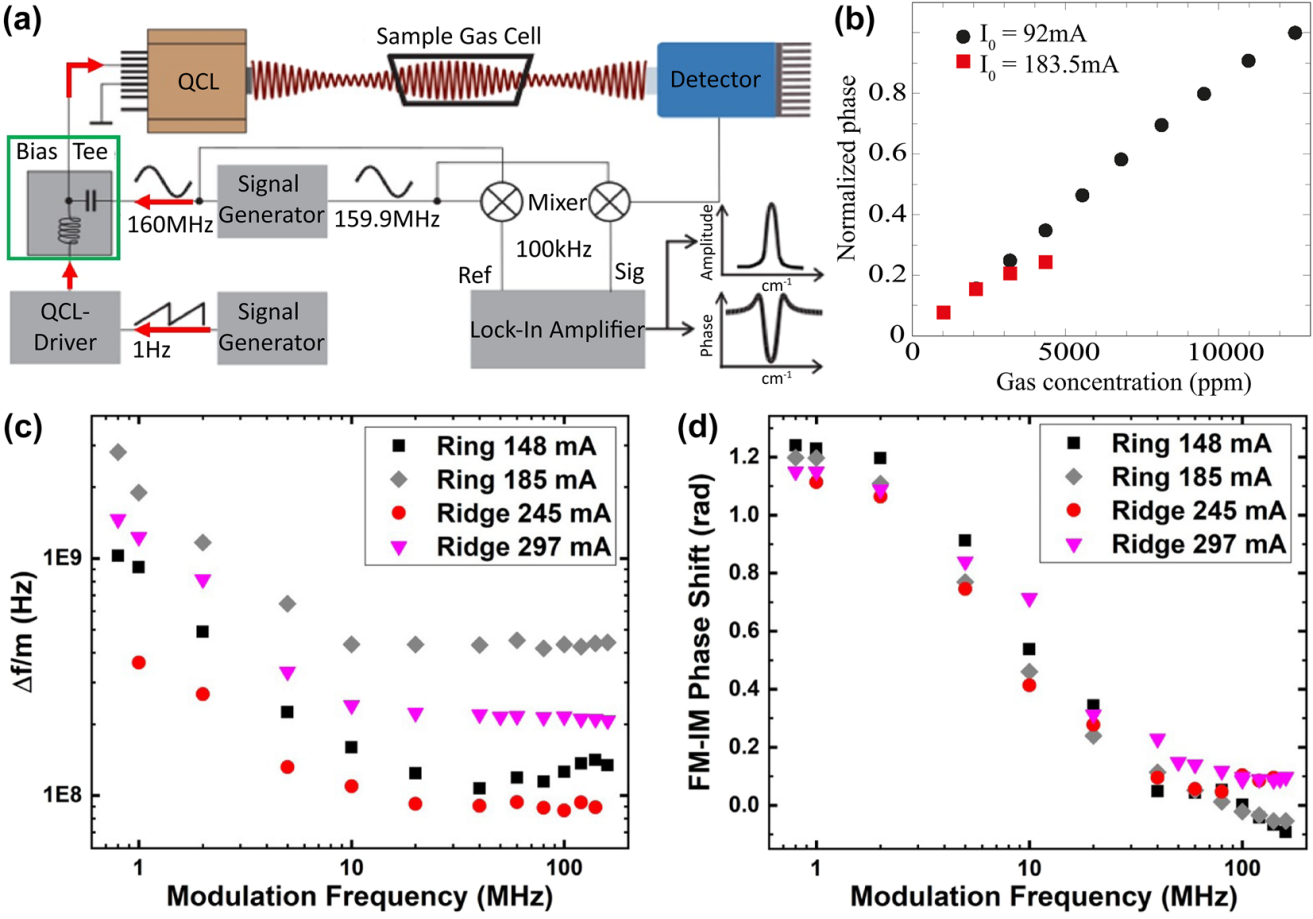
Modulation techniques and phase-sensitive dispersion spectroscopy with ring QCLs. (a) Sketch of the HPSDS setup. (b) The two identified operating regimes facilitate HPSDS sensing with two different dynamic ranges. (c) Comparison of the FM/IM ratio between ring QCLs and straight DFB QCLs. The increase below 20 MHz indicates the onset of thermal tuning. (d) Comparison of the FM/IM phase shift between ring QCLs and straight DFB QCLs. (a) Is reprinted from Ref. [92], with permission (CC BY 4.0); (b) is reprinted from Ref. [91], with permission (CC BY 4.0); (c–d) are reprinted from Ref. [92], with permission (CC BY 4.0).

Szedlak et al. were the first to implement HPSDS using ring QCLs, providing valuable insights into the modulation dynamics of these devices [91]. Figure 10(a) depicts the experimental setup. This study explored the relationship between the tuning parameters of the laser and a well-known absorption profile of CH_4_ in N_2_. Key characteristics such as the FM/IM ratio (the ratio of frequency modulation to intensity modulation) and the FM/IM phase shift were measured across a modulation frequency range from 1 to 150 MHz. The results demonstrated a relatively flat behavior from 20 to 150 MHz, with a marked increase in the FM/IM ratio below 20 MHz. This low-frequency behavior was attributed to the onset of thermal tuning, which serves as an important figure of merit for the laser’s response.

The characterization of the tuning properties laid the groundwork for subsequent HPSDS experiments. The study identified two distinct operating regimes, each corresponding to different bias current setpoints, as outlined in Figure 10(b). A low bias current exhibited a small FM/IM ratio, which resulted in an expanded dynamic range, a desirable attribute for HPSDS applications. Conversely, at higher bias currents, the FM/IM ratio and the phase shift increased, leading to a higher signal-to-noise ratio but at the expense of linearity. This dual-mode operation facilitated the optimization of the system, achieving detection limits of 16 ppm and 2 ppm in the two regimes, respectively.

Building on this work, Hinkov et al. conducted a comparative analysis between straight DFB QCLs and ring QCLs [92]. The findings revealed similar trends in the FM/IM ratio and phase behavior between the two device architectures, as shown in Figure 10(c) and (d), respectively. However, in the case of ring QCLs, a quasi-single-sideband modulation regime was observed at high modulation frequencies (100 MHz) and low bias currents. This discovery highlights the potential of ring QCLs for compact and versatile MIR gas sensing applications that leverage efficient modulation techniques.

The understanding of these RF modulation characteristics opens new avenues for the deployment of ring QCLs in advanced spectroscopic setups. The ability to precisely control and exploit the laser’s tuning dynamics enhances the sensitivity and selectivity of detection methods, thereby expanding the applicability of ring QCLs in fields requiring high-precision gas sensing.

### 5.2 Single-chip spectroscopy

For numerous applications, including environmental monitoring, industrial process control, and medical diagnostics, compact sensors in the MIR range are highly desirable. However, achieving this compactness while maintaining functionality has posed challenges, especially given that most miniaturization strategies in MIR sensing rely on discrete optical components. Conventional sensing systems generally consist of a separate light source, an interaction region where the light interacts with the analyte, and a detector to analyze the light after it has interacted with the substance. The drive towards on-chip solutions has motivated efforts to integrate these components within a single device, reducing complexity and increasing stability and portability.

In the realm of quantum cascade technology, integrating a laser and detector on the same chip has long been hindered by the inherent blue-shift of the QCL in detector operation. This challenge was addressed by Schwarz et al. with the introduction of bi-functional quantum cascade lasers/detectors (QCLDs) [93]. These devices leverage the same quantum cascade heterostructure for lasing and detection at the same frequency, thus enabling the fabrication of both components on a single chip. In these bi-functional QCLDs, the active regions for lasing and detecting share the same material and structure, simplifying the fabrication process and reducing cost. On-chip sensors using facet-emitting QCLDs have been successfully demonstrated, with the emitted light from the laser coupled into the adjacent detector via the facet.

One notable implementation of such devices was realized in liquid sensing, using a surface plasmon polariton (SPP) waveguide between the laser and detector [94]. The SPP waveguide confines the light and enables sufficient interaction with the liquid environment for sensing. However, due to their relatively short propagation lengths, SPP-based sensors are effective for liquid sensing but are not well-suited for gas sensing, which requires longer interaction paths.

**Figure 11:**
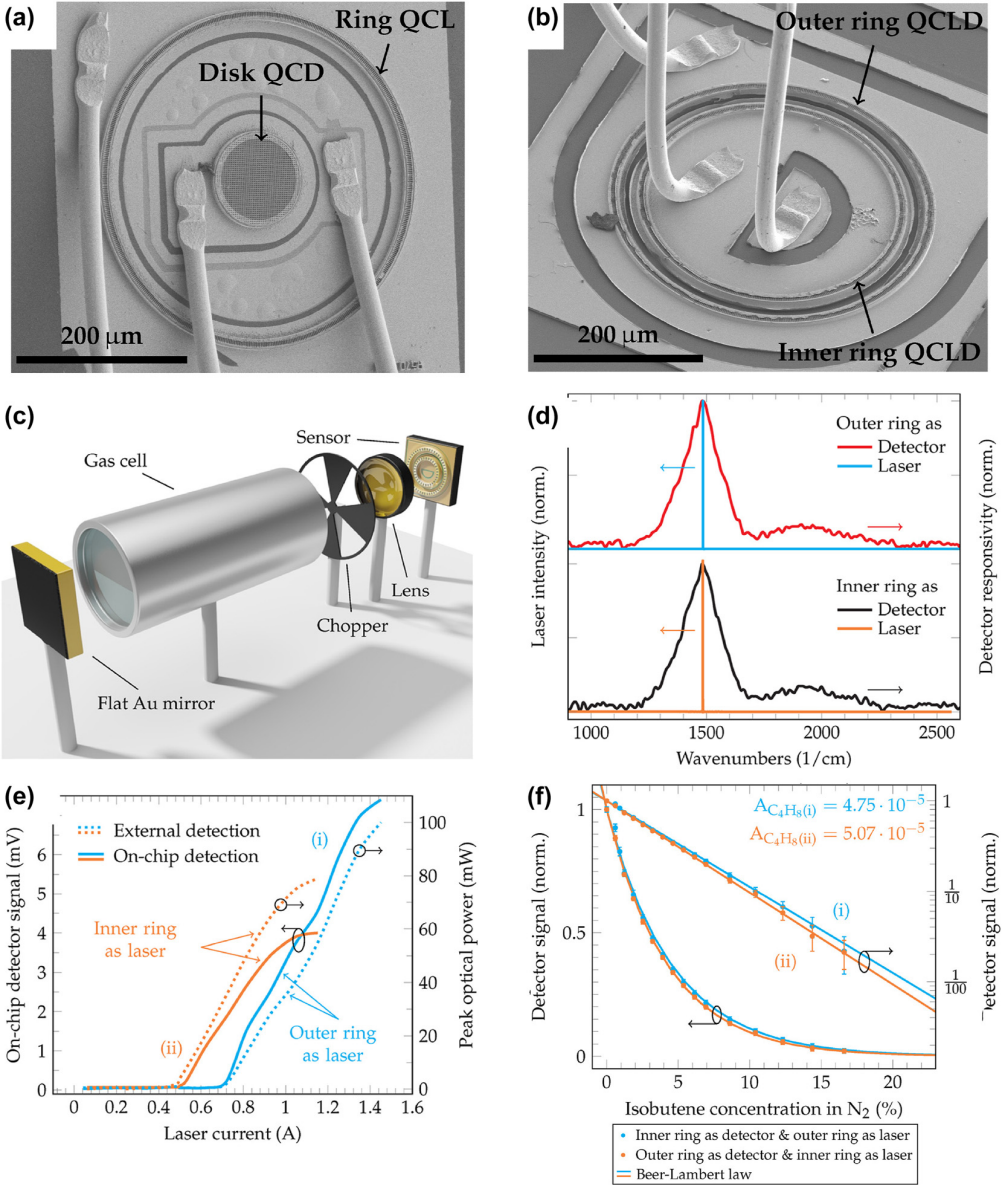
Monolithic ring laser and detector integration for gas sensing. (a) SEM image of the first surface-emitting and -detecting quantum cascade device. (b) SEM image of the commutable concentric ring QCLDs. (c) Sketch of the remote sensing setup. The emitted light from one ring is guided through the gas cell before it is back-reflected by a flat gold mirror and detected on the same chip by the other ring. (d) Laser and detector spectra of both rings with an optimal overlap around 1,500 cm^−1^. (e) LI characteristics of both rings recorded with an external (dashed) and on-chip (solid) detection scheme. The threshold currents of 0.48 A for the inner ring and 0.68 A for the outer ring correspond to threshold current densities of 4.8 kA/cm^2^ and 5.6 kA/cm^2^, respectively. (f) Proof-of-principle gas sensing measurement results of isobutene. (a–b) Are reprinted from Ref. [91], with permission (CC BY 4.0); (c–f) are reprinted from Ref. [56], with permission (CC BY 4.0).

In response to this challenge, attention was directed towards surface-emitting devices, such as ring QCLs, to provide a viable solution. Harrer et al. demonstrated the first bi-functional surface-emitting and -detecting device based on quantum cascade heterostructures [95]. As represented in Figure 11(a), this innovative sensor design consists of a surface-emitting ring QCL and a disk-shaped quantum cascade detector (QCD) positioned at the center. While the ring QCL achieves surface emission through a second-order DFB grating, surface detection in the disk QCD employs a squared metal hole grating to couple incident light to the detector element. The sensor was implemented in a setup where the emitted light from the ring QCL was directed through a gas cell and back-reflected by a flat gold mirror. The back-reflected light passed through the gas cell again and was detected by the disk QCD on the same chip. Proof-of-concept gas sensing experiments were performed at cryogenic temperatures.

However, the performance of this sensor was constrained by its inherent geometry. The ring QCL generates a ring-shaped farfield pattern, which, after passing through the optical system twice, is mapped onto a ring-shaped intensity distribution on the sensor chip. Hence, much of the intensity is missing the disk detector leading to suboptimal detection [91].

To address this limitation, Szedlak et al. developed a solution that employed two concentric ring QCLDs with slightly different diameters instead of one ring laser and a disk detector [56]. This improved design is depicted in Figure 11(b). The ring-shaped beam pattern is now directed onto a ring-shaped detector, offering a more efficient coupling mechanism and thus yielding superior sensor performance compared to the previous disk detector configuration. A sketch of the experimental sensing setup is outlined in Figure 11(c).

This work marked the first demonstration of a ring QCL functioning as a detector. In these detector devices, the DFB grating is used to couple surface-incident light into the active region. Both concentric ring QCLDs are capable of lasing and detecting, and their dual functionality was demonstrated by interchanging the roles of the outer and inner rings as laser and detector. This interchangeability is demonstrated by the corresponding spectra and LIVs depicted in Figure 11(d) and (e), respectively. The study further validated the performance of these on-chip detection schemes by comparing them with external detection setups, showing excellent agreement.

Moreover, the authors implemented two distinct DFB grating periods to achieve different emission wavelengths from the two rings without affecting the detector spectra. Using the same setup, where one ring emitted light through a lens and gas cell, which was then back-reflected and passed back through the gas cell and lens onto the second ring, the authors successfully differentiated between two gaseous analytes based on their absorption characteristics. By leveraging these dual wavelengths, the researchers achieved selective gas detection with a limit of detection of 400 ppm at room temperature, without requiring temperature stabilization and an absorption length of 20 cm. The corresponding gas sensing measurment of isobutene is outlined in Figure 11(f).

This successful implementation showcases the potential of ring QCLDs for remote sensing applications in compact, monolithic on-chip designs. The ability to integrate surface emission and detection capabilities, combined with the flexibility to achieve dual-wavelength operation, highlights the versatility of these devices for advanced MIR sensing technologies.

## 6 Ring interband cascade lasers

Interband cascade lasers (ICLs) represent a class of MIR semiconductor lasers that share the cascading nature of QCLs but differ fundamentally in their operating mechanisms [96], [97], [98], [99]. While QCLs rely on intersubband transitions within a single conduction band, ICLs employ interband transitions between the conduction and valence bands. This distinction results in ICLs exhibiting lower power consumption compared to QCLs, making them well-suited for compact sensing solutions where energy efficiency is crucial [100], [101], [102]. The surface-emitting ring concept, originally developed for QCLs, can also be extended to other laser platforms, including ICLs, enabling new design opportunities. Building on this, the introduction of ring ICLs, with their compact form factor and energy-efficient operation, has expanded applications to include portable and wearable sensing devices. Moreover, due to the interband nature of ICLs, it is feasible to realize VCSELs [103], [104], [105], unlike QCLs, which are restricted by the intersubband selection rule.

**Figure 12:**
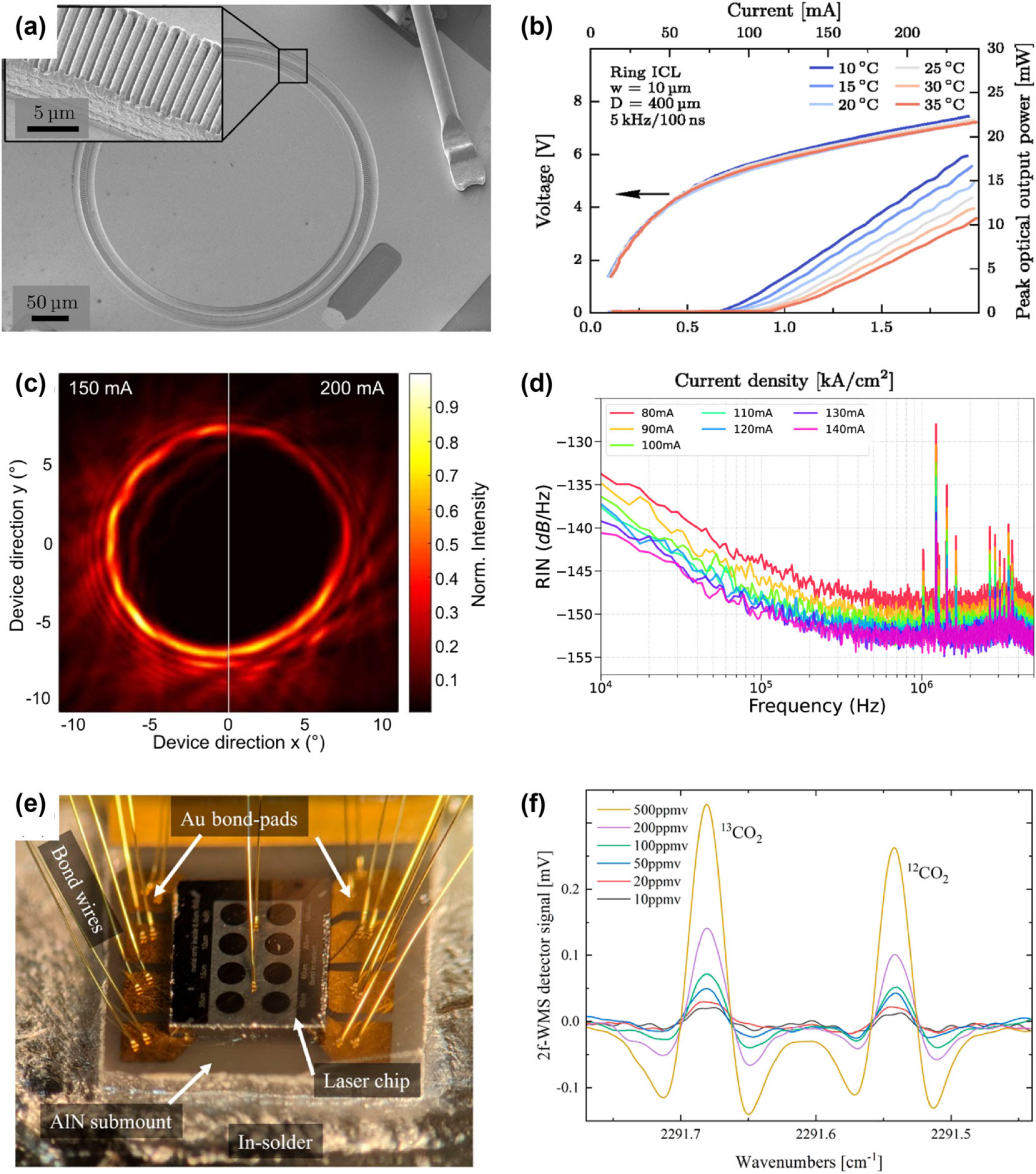
Characterization and applications of ring ICLs. (a) SEM image of the first ring ICL. The inset shows the second order DFB grating. (b) LIV characteristics in pulsed mode of the ring ICL. (c) Recorded farfield of a ring ICL without the characteristic interference rings. (d) Relative intensity noise of a ring ICL at different driving currents. (e) Photograph of an epi-side-down bonded array of 8 ring ICLs on an AlN submount. (f) Absorption spectra of CO_2_ isotopes, recorded with a CW ring ICL. (a–b) Are reprinted from Ref. [106], with permission (CC BY 4.0); (c) is reprinted from Ref. [107], with permission (CC BY 4.0); (d) is reprinted from Ref. [108], with permission (CC BY 4.0); (e–f) are reprinted from Ref. [109], with permission (CC BY 4.0).

Holzbauer et al. demonstrated the first ring ICL in 2017, marking a significant step forward in compact, surface-emitting MIR lasers based on interband cascade technology [106]. Figure 12(a) depicts the first ring ICL. These devices share several design principles with ring QCLs, including their surface-emitting configuration, but exhibit a crucial difference in the emitted polarization. In ring QCLs, the polarization is typically azimuthal due to the TM nature of QCL emission, while in ring ICLs, it is radial because of their transverse electric (TE) polarization. The authors achieved single-mode emission around 3.7 μm at room temperature with these devices.

One key design feature of their ring ICLs is the use of a second-order DFB grating, which is entirely filled with gold, unlike in ring QCLs, where the grating slits are filled with air. Consequently, only substrate emission was observed in the ring ICLs. As outlined in Figure 12(b), the reported device achieved a room-temperature pulsed optical output power of 15 mW, a threshold current density of 0.75 kA/cm^2^, and a slope efficiency of 104 mW/A. While the output power and slope efficiency exceeded those of interband cascade VCSELs, they were lower than those of edge-emitting DFB ICLs. Moreover, ring ICLs provide a unique advantage over interband cascade VCSELs due to their scalability, which enables increased output power through adjustments to the ring’s radius or width.

Building on this initial demonstration, Knötig et al. refined the fabrication and mounting of ring ICLs, achieving CW operation up to 38 °C [107]. Key improvements included reducing the waveguide width from 10 µm to 4 µm to suppress higher-order radial modes and implementing an epi-side-down mounting technique to enhance heat extraction. As a result, the CW output power at room temperature reached 6.4 mW, with a threshold current density of 0.6 kA/cm^2^ and a slope efficiency of 73 mW/A. The measured farfield intensity distribution showed a single ring pattern with an opening angle of approximately 15°, as illustrated in Figure 12(c). The absence of concentric interference rings suggests that the excited mode within the laser cavity is not the DFB grating mode, indicating a different coupling behavior in ICLs, likely due to their TE polarization.

In addition to these advancements, Marschick et al. provided a comprehensive noise characterization of ring ICLs, focusing on the bias-dependent intensity noise power spectral density (INPSD) and relative intensity noise (RIN) [108]. Employing a balanced-detection scheme, the researchers demonstrated shot-noise-limited operation between 100 kHz and 5 MHz. Notably, the RIN values (see Figure 12(d)) of ring ICLs were orders of magnitude lower than those of conventional straight DFB ICLs, likely due to the absence of facets in ring ICLs. This reduction in noise makes ring ICLs promising candidates for precision applications, such as interferometry and advanced spectroscopy, where a shot-noise-limited source is crucial.

Battery-driven applications particularly benefit from the low power consumption of ICLs. Furthermore, a small device footprint is not only desirable for compactness and energy efficiency but also reduces production costs. A smaller laser footprint directly enhances portability and system compatibility, enabling their integration into compact electronic systems, wearable devices, and miniature sensors. Moreover, miniaturization often leads to improved energy efficiency, as smaller device dimensions correlate with lower power consumption. This is advantageous for battery-powered or resource-constrained environments. By shrinking and densely integrating ring ICLs, substantial cost reductions can be achieved due to more efficient material usage and the ability to produce larger quantities of devices per wafer. Marschick et al. further advanced the miniaturization of ring ICLs by investigating the impact of reducing the ring diameter on the performance of the laser, specifically on threshold current density [109]. They identified an optimal radius of 80 µm, as smaller rings exhibited significantly higher threshold current densities. As presented in Figure 12(e), they employed an epi-side-down bonding technique on an AlN submount, enhancing heat dissipation and enabling CW operation. This mounting method also facilitated on-chip testing during the fabrication process, which is beneficial for industrial-scale production. Proof-of-concept gas sensing measurements of two CO_2_ isotopes demonstrated the applicability of this compact light source. Using wavelength modulation spectroscopy (WMS), the researchers improved sensitivity by an order of magnitude compared to conventional direct absorption spectroscopy. Figure 12(f) shows the WMS signal of the two CO_2_ isotopes. An absorption length of 10 cm allowed the authors to reach detection limits of 24 ppmv for ^12^CO_2_ and 13 ppmv for ^13^CO_2_.

These results highlight the potential of ring ICLs for various industrial applications, including environmental monitoring, process control, and atmospheric research, where compact and low-power sensors are essential.

## 7 Other approaches

In the quest for efficient surface emission in MIR and THz QCLs, various designs beyond ring QCLs have been explored, including DFB surface emitters, PC QCLs, circular grating QCLs, and random lasers, each with distinct advantages and limitations. DFB surface emitters have garnered attention due to their high output power and capability for CW operation at room temperature [28], [29], [110]. However, these emitters typically produce a wide, double-lobed farfield pattern, arising from the antisymmetric mode supported by their geometry [111]. To overcome this, modifications to the DFB grating have been shown to suppress the antisymmetric mode, favoring the symmetric mode and yielding a single-lobed farfield pattern, as demonstrated by Boyle et al. and Sigler et al. in the MIR [112], [113]. For THz QCLs, Xu et al. introduced graded photonic heterostructure gratings that similarly promote lasing in the symmetric mode, resulting in a well-defined, single-lobed farfield [114].

PC QCLs offer a more tailored approach, allowing for efficient surface emission with controlled beam divergence and specific spectral characteristics. The initial demonstration by Colombelli et al. involved MIR PC QCLs with air holes etched through the active region to enable the necessary optical feedback [27]. This structure was later adapted to THz wavelengths by Dunbar et al. [115] and subsequently enhanced by Loncar et al., who implemented it for optofluidic applications [116]. Further work by Chassagneux et al. examined how boundary conditions influence the performance of PC QCLs [117]. Large-area PC QCLs, as demonstrated by Yao et al., Wang et al., and Liang et al., achieved Watt-level output power but remain restricted to pulsed operation [118], [119], [120]. Yao et al. presented a substrate-emitting PC QCL capable of single-mode operation at room temperature, reaching Watt-level output power; however, this device operated exclusively in pulsed mode and exhibited a non-concentric farfield pattern [118]. Liang et al. introduced a PC QCL with a deep-etched buried grating that enabled low-divergence, single-mode emission without side lobes. However, this design was constrained by a maximum output power of below 200 mW [120]. Wang et al. subsequently improved the design, achieving Watt-level output power [119], but this laser exhibited multimode emission and a relatively wide farfield divergence of approximately 10°.

Circular grating QCLs, demonstrated by Liang et al., extend surface-emitting capabilities in the THz range by using concentric second-order gratings that yield single-mode emission with a collimated farfield [121], [122], offering a compact alternative to both DFB and PC QCLs. Xu et al. introduced a vertical-external-cavity surface-emitting-laser (VECSEL) configuration that employs an active quantum-cascade metasurface reflector and an output coupler, enabling vertical emission with high-quality beam profiles in the THz range [123]. The metasurface is composed of a sparse array of metal-metal ridges forming waveguide sub-cavities. Follow-up work demonstrated focusing metasurfaces, where the ridge width is spatially modulated both along and transverse to the ridges to improve power and beam quality [124], as well as electrically switchable polarization control [125]. Random lasers present a unique design approach, as introduced by Schönhuber et al., where randomly placed scattering elements within the laser cavity create frequency-independent feedback, enabling surface emission across a broader spectrum [78]. This configuration, in contrast to ring QCLs, supports broadband emission but has also been adapted for tunable single-mode output through a reconfigurable optical field that alters the active region’s permittivity [126].

Together, these diverse surface emission approaches expand the range of potential QCL applications across MIR and THz frequencies, with design trade-offs allowing customization based on specific power, beam quality, and operating mode requirements.

## 8 Conclusion and perspective

This comprehensive review has extensively explored the development, performance, and multifaceted applications of ring QCLs and, more recently, ring ICLs. Characterized by a ring-shaped cavity with a second-order DFB grating, ring QCLs achieve controlled surface emission, stable single-mode operation, and produce collimated, circularly symmetric beams. The successful demonstration of CW operation at room temperature has broadened the scope of ring QCL applications, while the advancement of microring QCLs points to scalable, miniaturized production and facilitates the possibility of large-scale manufacturing. Additionally, the extension of ring QCL technology into the THz regime has unlocked new opportunities in spectroscopy, imaging, and communication. Enhanced grating designs, including the integration of phase shifts, allow precise tailoring of farfield patterns, effectively addressing challenges like the inherent central intensity minimum. Furthermore, on-chip optical solutions, such as metasubstrates for beam collimation and OAM generation, have broadened the applications of ring QCLs. The advancement of integrated QCLD architectures, combining both emission and detection capabilities within a single chip, represents a promising pathway for creating compact, miniaturized sensing systems. Such architectures, especially in ring-based QCLDs, facilitate efficient coupling between emitted and detected signals, presenting valuable prospects for remote sensing and selective gas monitoring applications. The introduction of ring ICLs, with their inherent advantages of lower power consumption and compact form factor, has expanded potential uses in portable, battery-powered devices, and wearable sensing solutions.

Looking forward, the field of MIR sensing stands poised to benefit from several key advancements. The continued miniaturization and integration of ring-based laser and detector systems will facilitate the development of portable, high-sensitivity sensors. Thermally stable and wavelength-consistent operation at room temperature enhances the suitability of these devices for a wide range of industrial and environmental monitoring applications. Combining ring QCLs and ring ICLs with advanced modulation and detection schemes can further enhance the selectivity and robustness of spectroscopic systems, enabling real-time, interference-free analysis in complex gas matrices. The development of microring QCLs marks a significant step toward scalable, low-cost fabrication of surface-emitting QCLs with minimized power consumption. By leveraging innovative design and manufacturing techniques, these microring lasers promise to provide efficient solutions that can be widely adopted in various applications. This progress will broaden the reach of MIR sensing technologies in medical diagnostics, environmental monitoring, and industrial process control.

In summary, the advancements in ring QCLs and ring ICLs, particularly in terms of compact integration and spectral versatility, position these devices at the forefront of MIR sensing. With ongoing innovations in design and performance optimization, these devices hold promise for a new generation of robust, compact, and energy-efficient sensors, setting the stage for transformative applications in scientific and industrial domains.
